# Per- and polyfluoroalkyl substances (PFAS): persistence, toxicity, and emerging solutions

**DOI:** 10.1007/s00449-026-03317-7

**Published:** 2026-04-03

**Authors:** Aris Ismanto, Tony Hadibarata, Candra Wahyuningsih, Lilik Maslukah, Yusuf Jati Wijaya, Novia Safinatunnajah, Afidhah Puspita Widyani

**Affiliations:** 1 Department of Oceanography, Faculty of Fisheries and Marine Science, Diponegoro University, Semarang, Indonesia; 2 Environmental Engineering Program, Faculty of Engineering and Science, Curtin University Malaysia, CDT 250 Miri, Malaysia; 3 Master of Marine Science Program, Faculty of Fisheries and Marine Science, Diponegoro Universiy, Semaran, Indonesia; 4 Doctoral of Marine Science Program, Faculty of Fisheries and Marine Science, Diponegoro University, Semarang, Indonesia; 5 Center for Integrated Coastal Zone Management (ICZM Center), Diponegoro University, Semarang, Indonesia

**Keywords:** PFAS, Emerging contaminant, Persistence, Toxicity, Solutions

## Abstract

PFAS are found in soil, water bodies, food products, and the air. Per- and polyfluoroalkyl substances (PFAS) are synthetic organofluorine chemicals that have carbon-fluorine (C-F) bonds that make CF₃ or CF₂ moieties in their molecular structures. ause the C–F bond is so strong and stable that it makes them very hard to break down. Because PFAS is found in in water, soil, and biota, especially in fish and other aquatic species. Human exposure to PFAS has been associated with adverse health effects, including immune system suppression and increased susceptibility to infections. This review examines PFAS to elucidate their environmental sources, physicochemical characteristics, analytical detection methods, and control strategies. Current detection techniques for PFAS in water, soil, and biological matrices are critically evaluated, with emphasis on advanced analytical approaches. In addition, existing remediation technologies such as electrocoagulation, advanced oxidation processes (AOPs), high-pressure membrane separation, and adsorption are reviewed. Emerging remediation strategies, including metal–organic framework (MOF)-based adsorbents, mechanochemical degradation, and electron-beam treatment, are highlighted for their potential to overcome the limitations of conventional methods. Future methods for successful PFAS mitigation are also emphasized by discussing recent regulatory changes toward group-based PFAS management and sustainability-oriented policy frameworks.

## Introduction

### Background and motivation

The environmental challenge posed by organic pollution continues to be a concern, with the main contributors being untreated sewage (or wastewater), agricultural runoff, and improper disposal of organic material or waste. There are also many new pollutants such as polycyclic aromatic hydrocarbons (PAHs), endocrine disrupting chemicals (EDCs), microplastics and Per- and polyfluoroalkyl substances (PFAS) that originate from industrial processes, increased urbanisation, and inappropriate disposal of various types of materials, allowing them to find their way into the environment through water and soil [1, 2]. The industrial manufacture of organofluorine chemical compounds leads to the formation of PFAS which consist carbonfluorine (C-F) bonds with CF3 or CF2 structures [3]. PFAS compounds are widely distributed throughout soil and water sources, food products, and air environments because to the unique properties of carbonfluorine (C-F) bonds, which prevent them from breaking down and remain stable indefinitely [4]. PFAS fall into two primary categories: nonpolymeric and polymeric compounds. The backbone of fluorinated polymers is made up of carbon atoms that can be partially or fully replaced by fluorine atom to form high molecular weight compounds [5]. The nonpolymeric PFAS consist of two groups: perfluoralkyl substances which are fully fluorinated and polyfluoralkyl substances which are partially fluorinated along with their various functional group compounds including perfuoroalkyl sulfonic acids (PFSAs) and perfuoroalkyl carboxylic acids (PFCAs) and perfuoroalkyl sulfonamides (PFSNs) and fuorotelomer-based compounds (FT-PFAS including FTOHs) and semi-fluorinated aliphatic compounds such as alkanes and alkenes and their derivative compounds [6].

**Fig. 1:**
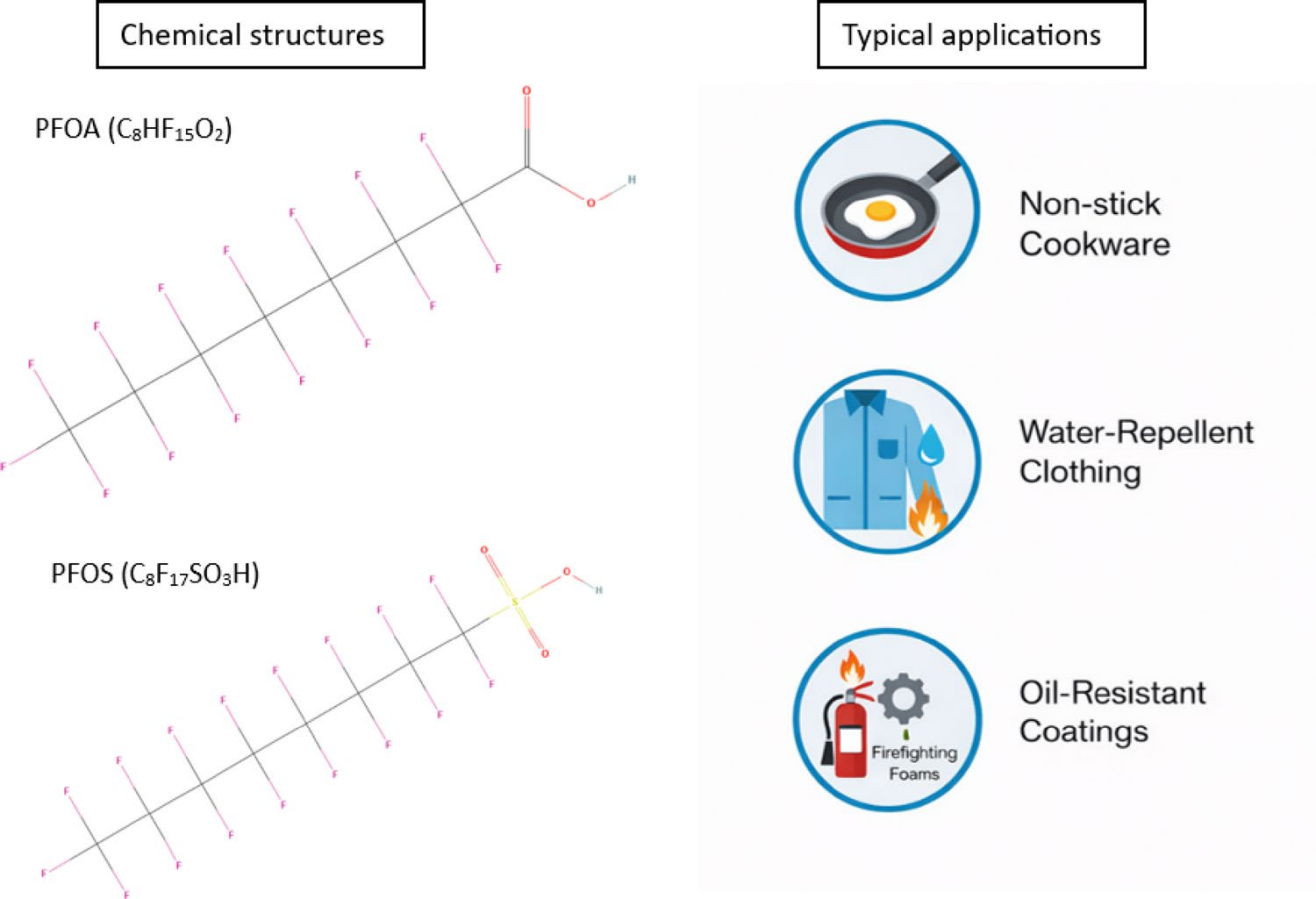
Representative PFAS structures and typical applications

PFAS molecule can interact with a variety of surfaces and fluids because they are generally amphiphilic, meaning they have two portion with distinct properties [7]. The first component is a fluorocarbon tail which is both hydrophobic (C-F bonds) and lipophobic. It is made up of a carbon chain that is saturated with fluorine atoms, making it extremely stable and difficult to react [8]. This component plays a role in providing water, oil and stain resistant properties to materials coated with PFAS. The hydrophilic head group, which is the second component control compound’s solubility and surface activity. Sulfonates (–SO₃⁻), like in PFOS (perfluorooctane sulfonate), are the two most prevalent functional groups. They are strongly acidic, negatively charged, and highly soluble in water, which makes them powerful surfactants. Another functional group is the carboxylate (–COO⁻), as in PFOA (perfluorooctanoic acid), which is slightly less hydrophilic than sulfonates but is still able to reduce surface tension and facilitate the spread of solutions on solid or liquid surfaces. The environmental behavior, bioaccumulation, and utility of each kind of PFAS in industrial and consumer applications are influenced by variations in the chemical characteristics of these two functional groups Ellis [9]. Figure 1. Representative PFAS structures and typical applications.

PFAS are often referred to as “forever chemicals” because they have physicochemical properties inherent in carbon-fluorine bonds that are resistant to degradation under certain environmental conditions [10]. As a result, many of these materials accumulate in the bodies of organisms that enter through the food chain, causing exposure to humans. Several studies mention that PFAS have a detrimental effect on human health, particularly the compounds PFOA and PFOS, which have been linked to developmental toxicity, liver damage, impaired fat metabolism, heart disease, immune system suppression, endocrine disorders, and kidney or testicular cancer [11].

Bioaccumulation of PFAS can pose risks to aquatic organisms and humans. These compounds accumulate in animal tissues and food crops, such as fish, vegetables, and grains, that live or grow in contaminated media [12–14]. The food chain enables human consumption of these foods which leads to toxic health problems. Research indicates PFAS exposure leads to bone damage and reproductive system problems and adipose tissue changes through specific biological receptor interactions [15, 16]. The long-term risks for both ecosystems and human populations become more evident because of these findings.

PFAS has spread throughout the environment because it accumulates in water and soil. Fish and other aquatic organisms have significant concentrations of PFAS in their bodies, which causes them to accumulate [17]. The food chain enables these chemicals to move from one organism to another through the consumption of these organisms. The death of organisms containing PFAS will lead to their environmental spread. The effects of high PFAS exposure levels differ between different species according to [13]. Human health faces two main risks from PFAS contamination through food and drink consumption and physical contact with contaminated environments [18]. The transfer of PFAS from contaminated areas to human populations becomes more efficient when plants and animals grow in these areas [14]. The human body absorbs PFAS through consuming contaminated food products and drinking contaminated water and by touching contaminated surfaces [19]. When exposed to PFAS, the human body suffers from two primary health issues: decreased immunity and increased susceptibility to disease [20]. The cardiovascular system faces damage from PFAS exposure which leads to stroke and diabetes and coronary heart disease [21] and respiratory tract infections [22] and gastrointestinal dysbiosis [23] and memory problems [12] and irregular menstrual cycles [15] as well as issues with excretion and musculoskeletal function [16, 22].

The worldwide distribution of PFAS creates a regulatory problem because these chemicals resistance o natural breakdown and the lack of effective waste treatment systems [24–26]. International environmental organizations support complete research methods to study PFAS ecotoxicological risks and create mitigation plans [11, 22]. The environmental persistence of PFAS and their toxic effects have led to stricter regulations on their use [5, 27]. The international community must prioritize PFAS as a critical environmental issue because it threatens both ecosystems and human health.

### Research gaps and review scope

The study assesses PFAS substances to determine their chemical characteristics, environmental sources, detection techniques, and control measures. PFAS infiltrate the environment through industrial products, textiles, food packaging, home items, and wastewater discharge, causing environmental contamination on a global scale ([5]; [28]). The substances persist in the environment while spreading through water systems and soil deposits and air circulation which results in their accumulation in living organisms [23, 29]. The extensive accumulation of PFAS in the environment leads to two major concerns: it causes harm to both wildlife and human health through endocrine disruption and organ damage [11, 22]. Through sophisticated analytical techniques, the study looks at existing PFAS detection approaches in various contexts [30, 31]. The review assesses current control and remediation systems which include electrocoagulation and advanced oxidation processes (AOPs) and high-pressure membrane separation and adsorption methods [24, 26, 32, 33]. Through its thorough analysis, the assessment lays the groundwork for the creation of environmental policies while offering extensive information regarding PFAS.

Despite extensive research on long-chain PFAS like PFOS and PFOA, critical gaps remain, especially regarding long-term toxicity of short-chain and emerging PFAS, mixture effects, and vulnerable populations. Because of the cost and complexity of the environment, remediation solutions frequently work poorly outside of lab settings, which limits their usefulness. Without clear scientific agreement, regulatory criteria differ by location, reflecting ambiguities in risk and exposure evaluations. This review synthesizes PFAS persistence mechanisms, compound-specific toxicity focusing on short-chain PFAS, and advances in remediation technologies and policies, addressing challenges from environmental behavior to health impacts and future research needs.

## Sources and environmental occurrence

### Industrial origins

The creation of the strongest single bond with carbon (C) in the 1930s, which substituted a fluorine atom (F) for the hydrogen atom (H) in the backbone (bond energy: 536 kJ/mol), marked the beginning of the development of PFAS [34]. This discovery led to the development of powerful synthetic chemical compounds called PFAS. This substance consists of 5000 different synthetic aliphatic substances which contain a hydrophobic backbone (tail group) in the form of a C atom with an F atom which partially saturates the C atom and has at least 1 functional group (R) such as carboxylate (–COO^−^), sulfonate (–SO^3−^), amine (–NH_2_), sulfonamide (–SO_2_NH_2_), alcohol (–OH) and phosphonate (–PO_3_^2−^) as hydrophilic end groups (head group) [31, 35]. In 1934 Polychlorotrifluoroethylene (PCTFE) was discovered by Fritz Schloffer and Otto Scherer as an initial form of PFAS molecules and was commercialized in the early 1950s. Roy J. Plunkett and Jack Rebok accidentally discovered polytetrafluoroethylene (PTFE), a white waxy solid branded as Teflon that was used in World War II, Apollo space missions to kitchen pans [3, 27, 36]. Additionally, PFAS are used as suppressors in aqueous film-forming foams (AFFFs), carpets, textiles, lubricants, and non-stick protective coatings, among other everyday [11, 37].

PFAS are a group of synthetic chemical compounds derived from several sources, including industrial products, consumer products such as waterproof textiles and food packaging, commercial household products, and contaminated drinking water [29]. The accumulation of PFAS-containing materials will become hazardous pollutants and pollute the environment. PFAS-coated food packaging materials have water- and oil-repellent characteristics and slow down food spoilage. These materials are widely used in the fastfood industry, especially in cutlery, popcorn bags, cupcake cups, instant noodle bowls, beverage containers, and parchment paper. As a result of being disposed of in landfills, disposable food packaging contaminates surface or groundwater [38].

PFAS are applied to household products that are waterproof, non-stick, stain-resistant, and easy to clean. Teflon (non-stick cookware), fast food packaging, and other home items are coated with polymer coatings that are resistant to heat and oil, which are made using PFAS ([33]; K. Singh et al. [39]. Waste from the manufacturing process and product degradation during use has the potential to enter the waste stream, which can impact water treatment systems or aquatic environments. Routes of PFAS contamination become more complicated when residential garbage and consumer goods waste are combined.

The environmental risks from Aqueous Film-forming Foam (AFFF) contamination persist because this firefighting agent is used at military bases and airports and airports. Significant levels of PFAS compounds, such as PFCAs, PFSAs, switterionics, fluorotelomers, and unknown novel compounds, are present in the firefighting foam AFFF. Multiple locations across the world show PFAS contamination in water and wastewater systems because of past PFAS product and raw material usage at industrial sites and military facilities [40]. The distance between contamination sources and their environmental impact determines how much PFAS compounds will accumulate in the environment [28]. The distribution of surface water contamination depends on multiple environmental factors which include temperature and precipitation and hydrological and hydrological conditions. The most commonly used PFAS products determine the extent of PFAS contamination between different countries. The United States and Europe have industrial and military facilities with PFAS contamination while Australian airports and military bases show PFAS presence in their water systems and Asia reports PFAS contamination in industrial site groundwater and river systems [28]. Through atmospheric transport, which allows these chemicals to move through the atmosphere until they settle in areas distant from their initial source, the distribution of PFAS expands [29]. Because the sources of the contamination are still unknown and pose risks to the public’s health and the environment, it is difficult to carry out effective mitigation and remediation.

### Occurrence in water, soil, air, and biota

Detecting environmental PFAS is crucial for two reasons: predicting the impacts of pollution and developing appropriate remediation strategies. The environmental persistence of PFAS together with their ability to move through different media types results in extensive contamination of water sources and soil and air and living organisms. Multiple research studies conducted in India and Colombia and Iran have identified PFAS contamination in river water and landfill environmental samples. The environmental stability of PFAS stems from their strong carbon-fluorine bonds which make them resistant to degradation. PFAS compounds are resistant to environmental degradation in water, soil, and air for prolonged periods of time because to the bonds that hold carbon and fluorine atoms together [41]. The natural breakdown of PFAS in environmental settings demonstrates to be extremely difficult when contamination exists and their movement becomes challenging to predict. Effective techniques for removing PFAS pollutants from drinking water must be developed by water treatment facilities [25].

The water-soluble characteristics of PFAS make water their main dissemination pathway. The release of PFAS into rivers and lakes and oceans results in their widespread distribution across many region beyond their original sources [42]. The hydrophilic and hydrophobic properties of PFAS determine its spread in aquatic environments. The aqueous phase contains short-chain PFAS but long-chain PFOS and PFOA tend to attach to organic matter and sediment particles which leads to accumulate in riverbeds [43]. The three compounds have been found in sediment samples that serve as repositories for PFAS, which are released into water during flooding or dredging operations. The US EPA has established 70 ng/l as the maximum allowed concentration of PFOA and PFOS and long-chain PFAS in drinking water [19]. The Bogota River contains twelve different PFAS compounds which were detected at levels between 0.06 and 0.52 µg/l. The three most prevalent PFAS compounds in the water were Perfluorohexanesulfonic Acid (PFHxS) at 0.52 µg/l Perfluorooctanesulfonic Acid (PFOS) at 0.24 µg/l, and Perfluorohexanoic Acid (PFHxA) at 0.10 µg/l [44]. The groundwater system will receive PFAS contamination through their mobility which threatens the drinking water supply of numerous communities [45].

PFAS mobilization spreads through soil and air in addition to water. PFAS contamination levels in plantation soil in Pakistan and China were 14.3–465 ng/g and 0.29–4.28 ng/g, respectively [46, 47]. Volatile precursors such as FTOH and perfluoroalkyl sulfonamides (FASA) will disperse throughout the atmosphere before degrading into stable PFAS such as PFCA and PFSA [48]. These volatile compounds are released into the atmosphere from industrial sites, waste disposal facilities, and the evaporation of contaminated water. Delhi, Mumbai, Chennai, Howrah, and Kolkata are among the places where PFAS have been found in the air, with concentrations as high as 820 pg/m^3^ [49, 50]. PFAS can spread across large areas that are far from the original pollution location thanks to atmospheric transport. PFAS enters terrestrial and aquatic ecosystems after settling in soil and water where it accumulates [51]. The use of AFFF as a volatile airborne firefighting foam at airports leads to PFAS contamination in surrounding areas.

Organisms that inhabit contaminated areas exhibit dominanting PFAS bioaccumulation patterns in their bodily tissues especially within aquatic environment [52]. PFAS shows different behavior than other persistent organic pollutants because it binds to proteins instead of accumulating in fatty tissues [53]. The accumulation of PFAS in fish and livestock and plants and human bodies pose serious health risks at high concentration. Plankton is at the top of the marine food chain, followed by small fish, giant fish, marine animals, and finally humans. The Ganges River fish contain PFAS at levels reaching 83.9 ng/g wet weight (w/w) according to [54]. The PFAS levels in apex predators reach their peak because they feed on smaller organisms that contain contaminants. Because it enables the material to travel from lower to greater quantities through animal eating, the process of biomagnification increases the dangers of PFAS exposure for both humans and wildlife [53].

### Global distribution and case studies

PFAS have been spotted in many regions at diverse concentrations. These dominants encompass aquatic environments, soil, waste disposal sites, potable water, and living organism. Furthermore, PFAS have been detected in human blood, hair, and breast milk specimens. Their pervasive existence result from the intensive utilization of PFAS in numerous products and their resistance to degradation. Consequently, PFAS accumulate in the food chain and the environment. Table 1 presents PFAS contamination data from several global regions at varying concentrations.

**Table 1:** Summary of PFAS contamination levels in different countries and matrices

Location	Samples type	Concentration	References
Southwest China near fluoropolymer manufacturing park. (industrial land use type)	Soils adjacent to a major PFAS production facility	355 *±* 65.8 ng/g (exceeds guideline values)	Fang et al. [55]
Oakey, Australia	Groundwater	4300 ng/L	Bräunig et al. [56]
Florida, USA	Biosolids	182–1650 ng/g	Thompson 57
India	Humans breast milk in Chidambaram, Chennai and Kolkata	< 1.66–335 pg/mL	Brusseau et al. [58]
South korea	Rice soils	0.12–13.9 ng/g	Kim Lazcano et al. [59]
Eastern China; largest vegetable production base (agricultural land use type)	Agricultural soils influenced by irrigation water, atmospheric deposition, and diffuse inputs	0.29–4.28 ng/g No/marginal exceeds guideline values	Zhang et al. [47]
Pakistan	Agricultural soil	14.3 465 ng/g	Baqar et al. [46]
Eastern United States	Underground drinking water	70–1645 ng/L	McMahon et al. [60]
Barcelona	Drinking water	30 ng/L	Cserbik et al. [4]
Pennylvania	Wastewater	155 ng/L	(Mroczko et al. (61)
Kenya	River surface water	6.7–26.17 ng/L	Chirikona et al. [40]
Netherlands	Drinking and surface water	0.4–1100 ng/L	Sadia et al. [62]
Iran	Solid waste landfill in Tehran	20.2 µg/L	Rasouli et al. [63]
Colombia	Bogota River	0.06–0.52 µg/L	Ramírez-Canon et al. [44]
India	WTP, surface water and groundwater in Chennai	0.1–136.27 ng/L	Koulini and Nambi [64]
India	Dolphin, fish and shrimp in Ganges river	0.093–83.9 ng/g w/w	Yeung et al. [65]

The extensive variations in PFAS concentrations documented in Chinese soils (Table 1) indicates significant variety in contamination sources and land-use characteristics rather than inconsistencies among studies. For example, Fang et al. [55] reported markedly elevated PFAS levels in soils adjacent to a major fluorochemical manufacturing park, representing a point-source industrial contamination scenario with long-term accumulation. In contrast, Zhang et al. [47] observed substantially lower PFAS concentrations in agricultural soils from China’s largest vegetable production base, where contamination is primarily associated with diffuse inputs such as irrigation water, atmospheric deposition, and biosolid application. These findings emphasize the significant impact of land-use type, proximity to emission sources, and exposure pathways on soil PFAS concentrations, highlighting the importance of contextualizing concentration data for evaluating contamination severity and regulatory compliance.

### PFAS in drinking water and food chains

**Fig. 2:**
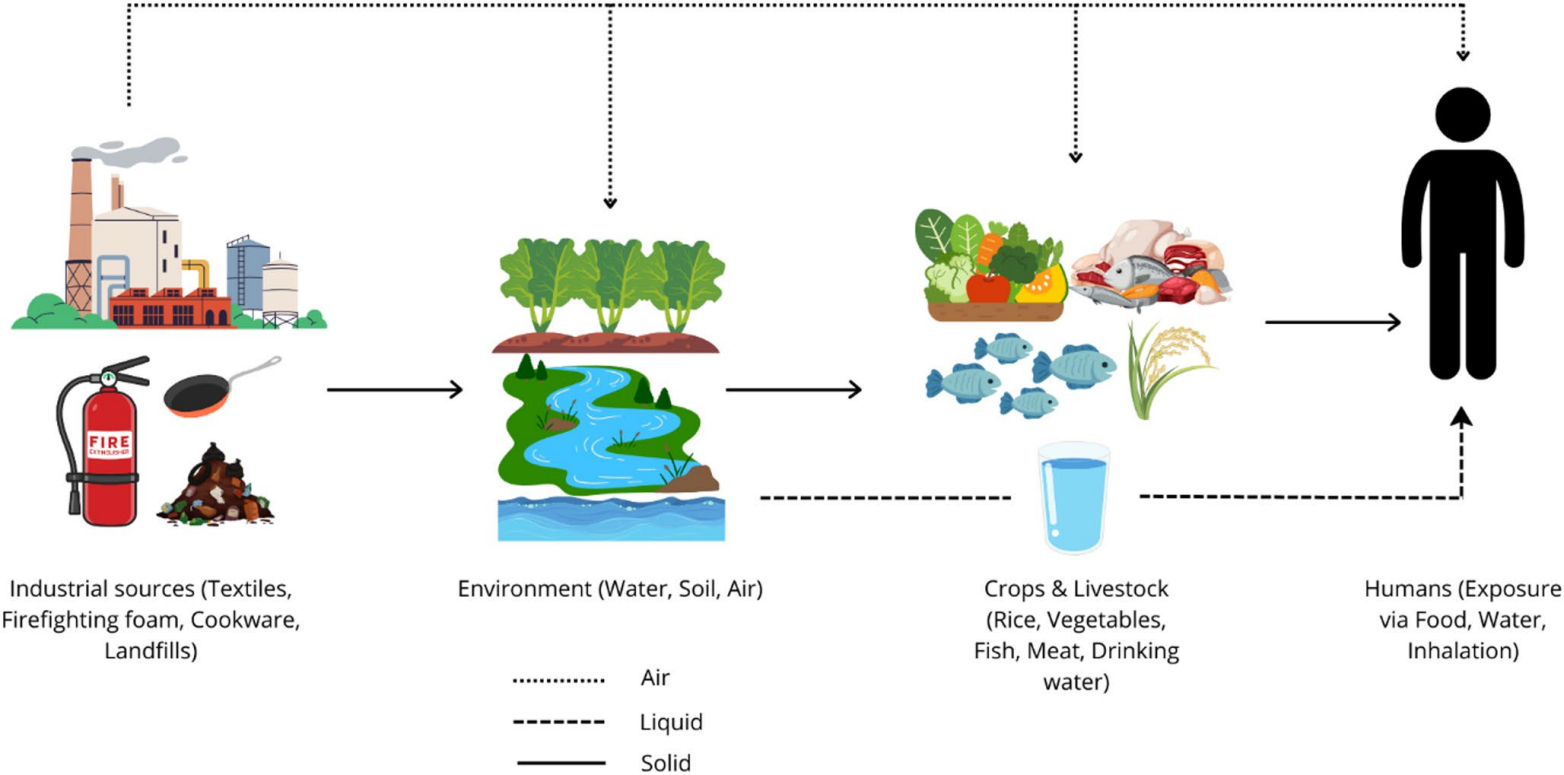
Conceptual diagram of PFAS transfer in the food web and human exposure routes

Drinking water contaminated with PFAS is a significant source of accumulation in the human body. In the Bogota River, Colombia, PFAS concentrations in surface water varied from 0.06 to 0.52 µg/l, with the predominant compounds being PFHxS, PFOS, and PFHxA [44]. This transpires as a result of anthropogenic and industrial activities that contaminate major water sources in densely inhabited regions. Several states in India have been detected with PFAS concentrations ranging from 10 to 120 ng/l due to industrial activities and the use of PFAS-based products [53]. Studies in Iran have reported PFAS contamination in landfill leachate, which has the potential to contaminate groundwater, which will then enter the drinking water supply system Rasouli et al. [63]. In the United States, regulations on drinking water quality are increasingly tightened due to the discovery of several PFAS-derived compounds in drinking water [66]. Figure 2. Conceptual diagram of PFAS transfer in the food web and human exposure routes.

Accumulation of PFAS in drinking water will infiltrate the bodies of organisms via the food chain (Fig. 2), may become polluted with PFAS by irrigation water or the application of biosolids containing PFAS [67]. The accumulation of these compounds will affect food safety due to health issues they pose to organisms and humans. PFAS contamination in Nigerian soil averages 15–40 ng/g, posing a risk of transfer to food crops [68]. In China, agricultural irrigation containing PFAS causes soil contamination of up to 0.29–4.28 ng/g [47]. Dong et al. [66] stated that testing conducted by the EPA and local industry detected PFOA and PFOS at concentrations of 110 ng/l in agricultural soil treated with biosolids. Agricultural products, as a fundamental food component, may serve as considerable indirect pathway for human exposure to PFAS.

PFAS can infiltrate the tissues of aquatic organisms, which are then ingested by humans. Concentrations of PFSAs, PFOSA, N-EtFOSE, PFCAs, and N-EtFOSAA in shrimp, fish, and dolphins in the Ganges River in India have been observed at levels ranging from 0.093 to 83.9 ng/g w/w [65]. Research conducted in in Lativia, Swedia, and United States, verified the presence of PFOS in fish tissues at values of 0.27–3.50 ng/g, 4600 ng/g, and 9620 ng/kg, respectively [69–71]. Studies in India stated that PFAS concentrations in human hair ranged from < 0.02 ng/g–3.78 ng/g with PFHxS, PFOS, and PFOA as the main compounds detected [72]. Breast milk in Kolkata, Chidambaram, and Chennai has been detected as containing PFAS at levels of < 1.66–335 pg/ml [58]. Bioaccumulation will escalate among end-users within the food chain. PFAS compounds will increase and accumulate in human tissues and organs due to the consumption of PFAS-contaminated food.

## Chemical properties and environmental persistence

### Unique C–F bond and implications for stability

PFAS are a class of synthetic chemicals with varying chain lengths and functional groups. The robust carbon-fluorine (C-F) bond confers significant stability and resistance to chemical and microbial degradation, leading to prolonged environmental persistence. PFAS exist in both solid and liquid forms, contingent upon their chain length. PFAS are characterized by one or more carbon atoms (C) bonded to a fluorine atom (F) instead of a hydrogen atom (H), setting them apart from non-fluorinated compounds and they contain at least one perfluoroalkyl group (C_n_F_2n+1_-R) [29]. Polyfluoroalkyl compounds have carbon atoms bonded to several fluorine atoms, but not all hydrogen atoms are replaced by fluorine. Perfluoroalkyl compounds have alkyl groups (carbon chains) in which all hydrogen atoms have been replaced by fluorine atoms (completely fluorinated).

The high ionization and electronegativity of fluorine result in very strong bonds in PFAS, with a bond dissociation energy reaching 531.5 kJ/mol [36]. Polyfluoroalkyl groups can convert into perfluoroalkyl substances in the environment by microbial biotransformation, chemical oxidation, and photodegradation. PFAS can be categorized into fluorotelomer-based polymers and non-polymers. Over time, polymeric PFAS side chains degrade into non-polymer PFAS that are persistent, bioaccumulative, and toxic. Fluoropolymers such as polytetrafluoroethylene (PTFE) or Teflon do not accumulate in biological systems [73]. However, the use of non-polymer PFAS surfactants (PFOA, PFOS) as emulsifiers, thermal degradation during product use, and environmental degradation can transform fluoropolymers into non-polymers that are hazardous to the environment. Common and hazardous PFAS include perfluoroalkyl carboxylic acids (PFCAs, PFOA) and perfluoroalkyl sulfonic acids (PFSAs, PFOS), which are characterized by hydrophobic fluorocarcone chains and hydrophilic carboxylic acid or sulfonic groups [29].

PFAS demonstrate lipophobic and hydrophobic properties owing to their fluorine atoms and strong C-F bonds [74]. PFAS compounds have solubility in both polar and non-polar solvents, resulting in the formation of different fluorine phases. The boiling and melting points of PFAS are directly proportional to their chain length. High vapor pressure makes PFAS more volatile and gaseous, thus increasing their mobility. At low vapor pressure, PFAS exist in liquid or solid form, contaminating surface water or groundwater [75].

### Classification: long-chain vs. short-chain PFAS

The occurrence and movement of perfluorinated compounds are contingent upon their amphiphilic characteristics, which are influenced by the length of the fluorinated chain [74]. Non-polymeric PFAS are classified as long-chain (> 7 carbons) and short-chain (4–7 carbons) [3]. Buck et al. [5] indicated that long-chain PFAS contain carbon chains exceeding seven for PFCA or six for PFSA, whereas short-chain PFAS consist of seven or fewer carbon atoms (PFCA) and six or fewer carbon atoms (PFSA). Long-chain PFAS including PFOA and PFOS have been replaced by short-chain PFAS (GenX, PFBS, PFBA, etc.) and ultra-short-chain PFAS (PFPrA, PFEtS, PFPrS, etc., which have 2–3 carbons) [50]. The difference in carbon chain length determines their environmental behavior and toxicokinetics. Fewer carbon chain reduces tissue persistence and toxicity (The Danish Environmental Protection Agency [76]). Hydrophobicity increases with C-F chain length, so long-chain PFAS are more readily adsorbed than short-chain PFAS, which are more water-soluble [77, 78]. Veciana et al. [26] stated that PFSA is more hydrophobic than PFCA due to the additional C-F bond.

Long-chain PFAS exhibit a strong affinity for blood proteins and bodily tissues, potentially accumulating in organisms with a long half-life (K. Singh et al. [39]. These compounds also enhance the potential for bioaccumulation within the food chain [53]. Short-chain PFAS have increased water solubility and minimal protein binding, facilitating its rapid infiltration into aquatic systems and subsequent contamination of surface and groundwater [67]. Although the level of bioaccumulation in organic fractions is lower than long-chain PFAS, their persistence is higher because they are difficult to degrade and increase the distribution of pollution [53]. Essentially, long-chain and short-chain PFAS not only determine toxicity to organisms and environmental impact, hence presenting dangers to humans and ecosystems (Table 2).

**Table 2:** Comparison of long- vs. short-chain PFAS: structure, behavior, toxicity

Category	Long-chain PFAS	Short-chain PFAS
Structure	C > 7 (perfluoroalkyl carboxylic acids, PFCAs), C > 6 (perfluoroalkyl sulfonic acids, PFSAs)	C *≤* 7 (perfluoroalkyl carboxylic acids, PFCAs), C *≤* 6 (perfluoroalkyl sulfonic acids, PFSAs)
Persistence in environmental	Strongly resistant to degradation	Also resitant, but more dispersive
Amphiphilicity (water solubility)	Hydrophobic, less transport in water, more adsorp in organic matters	More hydrophilic, mobile to groundwater and surface water, contaminated water and soils
Bioaccumulation	Strong protein-binding (blood) increased bioaccumuation in food chain and organisme	Weak protein-binding, low bioaccumulation but continuous exposure because the high mobility in environment
Representative compounds	PFOA (C8), PFOS (C8), PFNA (C9), PFDA (C10)	PFBS (C4), PFHxA (C6), PFPeA (C5)
Human half-life	PFOS almost 5 years; PFOA ranges between 2–4 years	PFBS 1 month; PFHxA less than 1 month
Toxicity	Well-documented hepatotoxicity, immunotoxicity, endocrine disruption, and carcinogenicity (kidney, testicular)	Documented hepatotoxicity and immunomodulatory effects at defined doses in animal and in vitro studies; lower potency than long-chain PFAS but measurable effects under repeated exposure
Toxicological endpoints	NOAEL/LOAEL established for multiple endpoints (liver, immune system)	NOAEL values reported for liver effects in animal studies (e.g., PFBS, PFHxA); emerging evidence of immunotoxicity from recent experimental studies (2024–2025)
Regulation	Phased out or restricted in many countries	Widely used as replacements; increasing regulatory attention

### Mobility in environmental matrices

The mobility of PFAS in environmental matrices is affected by physicochemical properties, specially the length of the carbon chain and the attached functional groups. The distribution of PFAS in soil and groundwater occurs through sorption, leaching, and partitioning mechanisms. The hydrophobicity of PFAS contributes to the sorption process of compounds into the soil through interactions with soil particles [79]. The hydrophobic interaction between PFAS and the solid phase of the soil is influenced by the ionic strength of the soil solution from the effect of salting or polyvalent cations in the soil that neutralize the negative charge on the headgroup, resulting in an interaction of the hydrophobic PFAS tail with the uncharged soil surface [29]. Electrostatic interactions are based on the charge on a compound’s functional group. The carboxylate and sulfonate groups in PFOA, PFOS, and 8 − 2 FtS are anionic, the amine and sulfonamide groups in PFOAA are cationic, and FtSaB is zwitterionic. Soil contains aluminosilicate or phyllosilicate minerals that have negative ions, which cause the attachment or sorption of positively charged PFAS (cationic or zwitterionic) [80]. Negatively charged PFAS, including PFAAs, exhibit minimal adsorption on soil or are adsorbed in negligible quantities [77].

Leaching from the soil surface to the subsurface and groundwater predominantly involves short-chain PFAS, which exhibit greater solubility in water. Jahn et al. [81] reported accumulations of PFOS and PFOA of 70–170 ng/l and 1,000–1,300 ng/l, respectively, following 50 and 10 years of irrigation construction, respectively. Accoring to Kim et al. [82], 355 out of 628 wells and springs in Bennington, Vermont, surpassed the maximum PFOA limit (20 ng/l) between 2016 and 2019, attributable to the operation of a Teflon plant from 1978 to 2002. Factors influencing PFAS leaching and distribution include distance, depth, biosolids (fertilizer/organic solid waste) application rate, irrigation, and soil characteristics [29]. Total PFAS concentrations in groundwater at the AFFF site decreased with increasing distance. Vertically, PFAS concentrations varied with increasing depth. PFAA concentrations at a depth of 0–2 m, range from 2000 to 10,000 ng/g, whereas at 4 m falls below the detection limit. However, with a groundwater flow rate of 21 m/year, PFAS will migrate quite rapidly to the lower layers [83]. The use of biosolids will increase the variability of PFOS and PFOA transport in the soil. Irrigation development around PFAS contamination sites will accelerate the distribution of PFAS into diverse environments [81].

Partitioning PFAS among many phases, including as water, soil, and biota, enhances bioavailability and accumulation within ecosystems [51]. PFAS partitioning is regulated by the equilibrium partition coefficient of a chemical and predicts its mobility. Long-chain PFAS (> C6) exhibit greater adsorption to soil compared to short-chain PFAS, which preferentially partition into the aqueous phase [77]. GenX and ADONA have lower Kd values ​​than dominant PFAS contaminants such as PFOA, PFOS, and PFHxS, thus increasing accumulation due to their higher mobility [84].

### Transformation pathways and degradation resistance

**Fig. 3:**
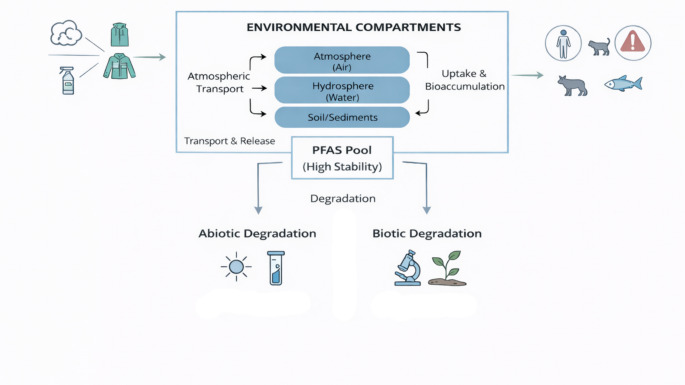
Environmental fate model of per- and polyfluoroalkyl substances (PFAS) showing their mobility, transformation, and persistence across air, water, soil, and biota. Abiotic transformation includes photocatalytic and electrochemical processes, while biodegradation involves limited microbial-mediated defluorination, mainly observed under laboratory conditions

The carbon-fluorine (C-F) bond, possessing a bond energy of roughly 485 kJ/mol is the strongest bond in organic chemistry [85]. The abiotic and biotic mechanisms of transformation and resistance to PFAS degradation have been investigated (Fig. 3). Abiotic PFAS remediation methods such as activated carbon adsorption, ion exchange, oxidation, and high-temperature incineration, have numerous disadvantage: they are costly, energy-demanding, and ineffective for degrading persistent pollutants [86, 87]. Khan [88] reported that these procedures generate harmful byproducts and just relocate toxins to different habitat. Cleavage of the carbon-fluorine (C-F) bond, or defluorination, can be carried out by microorganisms and their enzymes. Microbial consortia produce enzymes that degrade xenobiotics in an eco-friendly manner, and with the possibility for mineralization and minimal hazardous byproducts generation [89–91].

Microbial deflourination mechanisms are classified into three categories: reductive, hydrolytic, and oxidative deflourination, which are affected by enzyme systems, redox conditions, and the chemical structures of various fluorinated compounds (Hu and Scott [92]; [88]). Bacterial genomes encode a spectrum of enzymes, namely dehalogenases, monooxygenases, dioxygenases, and reductases, which work to break C-F bonds [92]. In addition, bacteria can adapt quickly to new environments, including anthropogenic pollutants, by modifying metabolic pathways. Bacteria will utilize PFAS as a source of carbon and energy for cell division and the synthesis of secondary metabolites [93]. *Labrys portucalensis* F11 isolated from contaminated soil can metabolize 90% of PFOS in 100 days. *Pseudomonas parafulva* strain YAB1 defluorinated PFOA up to 48% (Yi 94), *Pseudomonas aeruginosa* strain HJ4 only degraded PFOS in small amounts [95], *Gordonia* sp. strain NB4-1Y degraded 6:2 Fluorotelomersulfonic acid (6:2 FTSA) up to 88% [96], and *Pseudomonas fluorescens* DSM 83,413, *Pseudomonas butanovora*, *Pseudomona soleovorans*,* Mycobacterium vaccae* JOB5 degraded 6:2 Fluorotelomer alcohol (6:2 FTOH) up to 80–100% [97].

The abiotic and biotic degradation of PFAS results in hazardous metabolites, specifically simpler yet persistent PFAS compounds. This poses a considerable barrier in determining the appropriate PFAS degradation technology for recalcitrant and hazardous compounds [98]. The PFAS degradation process leads to full mineralization, producing HF, H_2_O, and H_2_O_2_ [99]. Abiotic treatment results in incomplete mineralization, while laccase, lignin peroxidase, and manganese peroxidase enzymes produce partial defluoronation of perfluoroalkyl acids [86, 100, 101]. The oxidative defluorination mechanism in enzymes also produces toxic and persistent substances, requiring post-treatment for complete mineralization [88]. A synergistic strategy integrating abiotic and biotic techniques is essential to enhance remediation efficacy and mitigate PFAS accumulation in the ecosystem.

## Toxicity and health effects

### Human exposure routes

Multiple primary exposure routes exist for humans to encounter PFAS which leads to elevated health risks because these substances exist throughout different environmental settings. Contamination of drinking water by PFAS constitutes the principal exposure pathway that researchers have thoroughly investigated because to its potential health risks. Water supply contamination occurs when industrial facilities and firefighting foam applications and municipal water systems discharge pollutants into potable water [102]. Rainwater contains PFAS contaminants which exceed U.S. EPA limits when it comes from local pollution sources [103] The body receives PFAS exposure through two essential routes which include food consumption. Studies indicate that food products sourced from industrial zones and contaminated locations harbor PFAS pollutants. Consumption of fish and other animals from polluted streams, as well as crops irrigated with PFAS-contaminated water, results in dietary exposure (Roth et al. 104). The western diet which includes more contaminated foods will increase the risk of health problems [105].

Residual exposure pathways are present via non-food objects, such as dust and consumer products. The accumulation of PFAS in indoor environments constitute significant exposure pathways because these chemicals infiltrate house via consumer products and building materials and furnishings. The exposure route affects spaces that use treated materials extensively including daycares and workplaces [106]. Consumer products including non-stick cookware and water-repellent clothing and cleaning solutions contain PFAS as their main ingredient. People develop PFAS exposure through skin contact and mouth ingestion when they touch their hands to their face. The use of PFAS in cosmetics and other personal products results in increased human exposure [107].

### Bioaccumulation and biomagnification

The synthetic chemicals known as PFAS show strong abilities to build up in living organisms which leads to dangerous accumulation of these substances throughout aquatic and terrestrial food chains. The synthetic compounds PFAS stay in aquatic systems while building up in different species which creates major problems for the entire food chain. The aquatic environment exhibits the highest levels of bioaccumulation via benthic macroinvertebrates, such as worms, mussels, and snails, due to their distinct habitat absorption processes and differing clearance rates (Yun et al. 108). The bioaccumulation factors of these organisms exceed those found in water column organisms. The food chain transfer of PFAS substances leads to contamination of fish and crustaceans and marine mammals which are at the top of their respective food chains [109]. The introduction of PFAS into the food web occurs via many channels, as these compounds are present in biofilms that serve as primary food sources [110]. The transfer of PFAS from aquatic environments to terrestrial ecosystems occurs through biological pathways. The emergence of aquatic insects from water bodies enables them to carry PFAS which then enters terrestrial food chains and affects birds and spiders that feed on these insects [111, 112]. Earthworms which exist at the bottom of the terrestrial food chain show evidence of PFAS accumulation which suggests PFAS can move from soil to upper food chain levels [52]. The concentration of PFAS substances grows more significant at each successively higher trophic level in food webs. The PFAS concentration levels in fish and their predators exceed those found in plankton and invertebrates which occupy lower positions in the aquatic food chain [110]. The bioaccumulation potential of long-chain PFAS surpasses that of short-chain PFAS due to their distinct physical and chemical properties, which influence the absorption and storage of these compounds by organisms [113].

### Documented health impacts

The ecological pollutants PFAS and PFAS have been demonstrated to induce many health complications due to their persistence in the environment, resulting in toxic effect that contribute to immunotoxicity and reproductive disorders, carcinogenesis, and hormonal system disruption. PFAS compounds create major damage to the human immune function. Scientific studies show that PFAS compounds lead to weaker vaccine responses in people of all ages. The immune system becomes less capable of fighting infections and cancer development because PFAS substances create immunotoxic effects [114, 115]. The reproductive system of women experiences severe damage because of PFAS exposure. Research shows that PFAS exposure leads to the development of polycystic ovary syndrome and premature ovarian failure and endometriosis and reproductive system tumors. The compounds create hormonal imbalances through their interference with the hypothalamic-pituitary-gonadal (HPG) axis and their impact on ovarian processes including folliculogenesis and steroidogenesis. The disruption of hormonal systems occurs because PFAS substances bind to serum proteins and hormones which results in changes to their transport and elimination patterns [15, 116, 117]. Previous study indicated that people who encounter PFAS face higher chances of developing specific types of cancer. Research findings show that PFAS exposure creates links to specific cancer types which include breast cancer and ovarian cancer. PFAS compounds make ovarian cancer cells resistant to carboplatin chemotherapy which demonstrates their dual role in cancer development and treatment resistance [118]. The primary evidence of endogenic disruption from PFAS exposure appears through their effects on hormonal systems and thyroid gland function. The chemical structure of PFAS allows them to act as endocrine disruptors which disrupts natural hormonal pathways. The reproductive system experiences disruption because PFAS compounds disrupt ovarian hormone production and create irregular menstrual cycles which result in premature menopause and irregular menstrual periods [119, 120]. Table 3 presents an overview of PFAS-associated health effects related with corresponding exposure levels. It is crucial to recognize that these effects differ significantly among various PFAS compounds. Long-chain PFAS, such as PFOS and PFOA, consistently demonstrate stronger immunotoxicity, including the suppression of antibody responses, altered cytokine production, and reduced vaccine efficacy, as evidenced by both epidemiological and animal studies. In contrast, PFHxS generally exhibits moderate immunotoxic effects, showing evidence of immune cell dysregulation but with lower potency than PFOS and PFOA Pierozan et al. [121], Tang [122].

**Table 3:** Summary of PFAS-associated health effects and corresponding exposure levels

Health effect	Associated outcomes	Exposure level/evidence basis	Key references
Immunotoxicity	Reduced vaccine responses (tetanus, diphtheria) Lower antibody production Impaired innate & adaptive immunity	Elevated serum PFAS levels correlated with weaker antibody responses in humans Animal studies show immune suppression at environmentally relevant doses	Bline et al. [123], Pappalardo [115]
Reproductive Disorders	PCOS Endometriosis Premature ovarian failure Disrupted ovarian hormone production	Epidemiological associations at typical environmental exposure levels Mechanistic studies show disruption of HPG axis and steroidogenesis	Ding et al. [15], Qu [117]
Cancer	Testicular and renal cancer Potential breast cancer link Chemotherapy resistance in ovarian cancer	Elevated cancer risks observed in populations exposed through contaminated water Mechanistic studies indicate tumor progression effects	Li [9], Rickard [118]
Hormonal Disruption	Thyroid hormone imbalance Altered metabolic regulation Shortened breastfeeding duration	Endocrine disruption observed across a range of PFAS serum concentrations in human cohorts Thyroid effects seen in both adults and children	Ask et al. [124], Timmermann [125]

Recent studies indicated that short-chain PFAS, including PFBS and PFHxA, can influence immune cell function in experimental conditions, primarily via mechanisms related to oxidative stress and modified immunological signalling pathways. Nevertheless, their immunotoxic potency seems to be diminished and is less thoroughly described owing to the lack of long-term exposure data. These compound-specific variations underscore the necessity of interpreting immunotoxicity in the context of chain length, functional groups, and exposure duration, rather than generalizing across all PFAS.

### Toxicokinetics and sensitive populations

PFAS are extremly persistent chemicals that raise significant concerns regarding toxicokinetics and pose specific risks to sensitive groups, including infants and pregnant women. PFAS compounds like perfluorooctane sulfonate (PFOS) and perfluorooctanoic acid (PFOA) have long biological half-lives, often several years, in human bodies due to their strong binding to blood proteins and slow elimination processes. For example, perfluorooctanoic acid (PFOA) has a half-life ranging from 1.48 to 5.1 years, while perfluorooctane sulfonic acid (PFOS) ranges from 3.4 to 5.7 years in humans [126]. These long half-lives contribute to their bioaccumulation in the human body. The half-life of PFAS compounds with shorter chains and fluoroethers extends from PFOA and PFOS but remains relatively long according to [127]. The compounds disseminate over multiple organs in the body that possess elevated levels of phospholipids, such as the liver and kidneys. PFAS compounds accumulate in the placenta during gestation due to alterations in maternal biochemistry [128, 129]. The Vulnerability of Sensitive Populations affects pregnant women and their infants and newborns. The fetus receives direct exposure to PFAS compounds through placental transfer which leads to negative pregnancy results for pregnant women. Research indicates that PFAS substances build up in placental tissue which causes structural changes and functional problems that lead to placental inefficiency and pre-eclampsia and low birth weight [130, 131]. The transfer of PFAS from mother to child during pregnancy creates a major health risk because these compounds move through the placenta and appear in breast milk. Newborns develop high levels of PFAS contamination because of this process. The efficiency of maternal-to-infant PFAS transfer depends on the specific chemical structure and characteristics of certain PFAS isomers [132, 133].

Some PFAS derivatives commonly found in drinking water have varying half-lives. PFOA (3.14 years), PFOS (3.36 years), PFNA (2.35 years), and PFHxS (8.30 years) [134]. Long half-lives can increase the effects of cumulative exposure. The volume of distribution of PFAS ranges from 0.19 to 0.43 L/kg and can accumulate in various organs, thus affecting liver and immune function [134]. Pregnant women and infants are vulnerable due to physiological changes that can increase toxicity. PFOS levels in pregnant women and babies were 6.17 ng/ml and 4.85 ng/ml, respectively [135]. Breast milk in Kolkata, Chidambaram, and Chennai has been detected as containing PFAS at concentration ranging from < 1.66–335 pg/mL [58]. Contamination transmitted through breast milk will increase the concentration of PFAS in the infant’s body. PFAS passes through placenta, exposure to the fetus in the womb results in fetal growth retardation and low birth weight [135, 136].

**Fig. 4:**
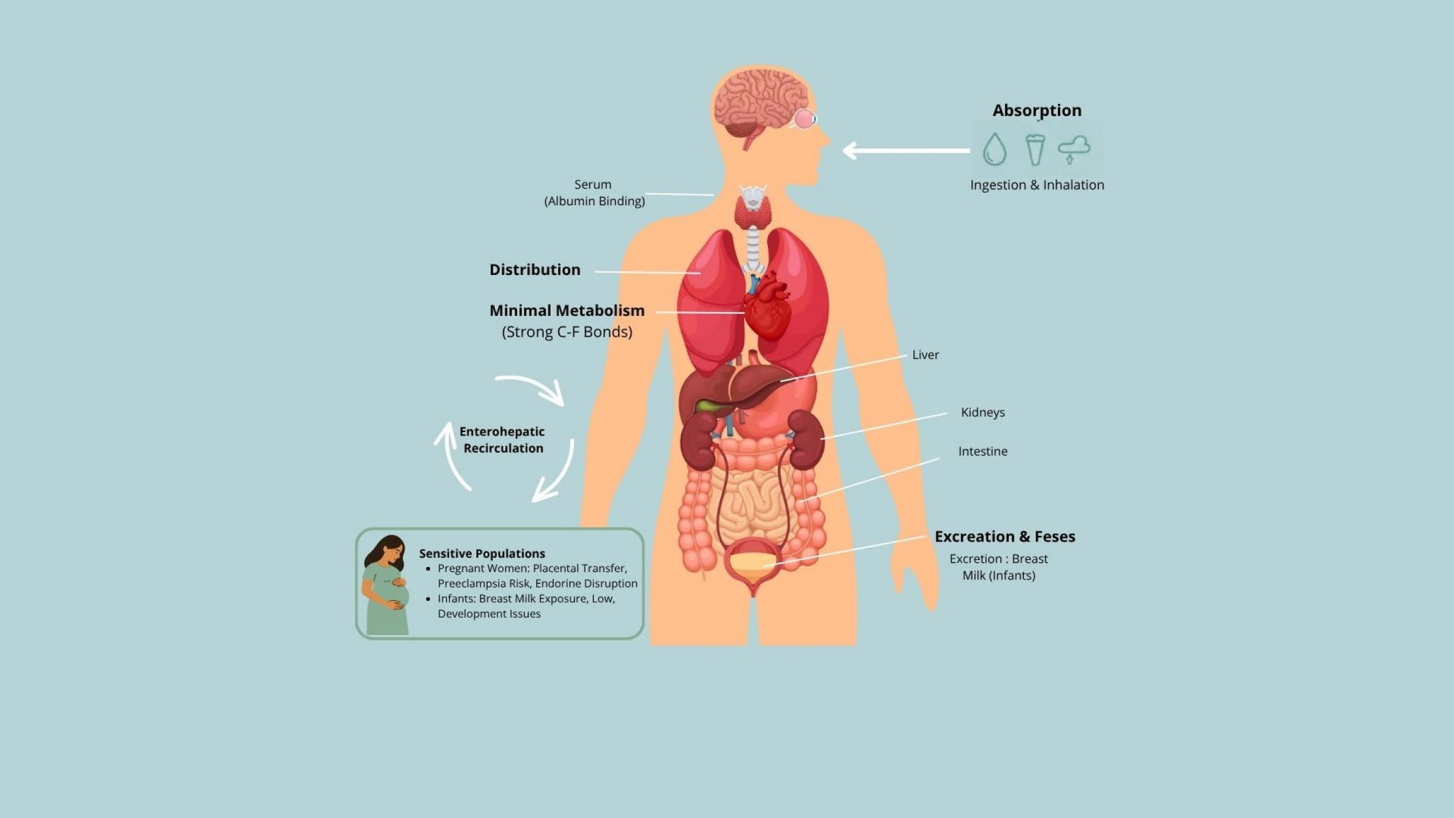
PFAS ADME (absorption, distribution, metabolism, excretion) schematic in humans

The mechanisms of absorption, distribution, metabolism, and excretion of PFAS in humans occur through complex processes (Fig. 4). PFAS absorption transpires via multiple pathways, including dermal contact, ingestion of contaminated food, and ambient exposure. High persistence results in the presence of PFAS in various media (Mišl’anová and Valachoviˇcová, 2025). The distribution of PFAS accumulated in the human body will enter the liver and brain tissue, especially in the hippocampus, which shows higher concentrations [137]. PFAS with longer carbon chains show greater accumulation [138]. The metabolic process of PFAS is limited to the human body. MRP1 and OATP2B1, along with ASCT1 and P-gp, have been identified as transporters involved in the dynamic transfer of perfluoroalkyl and polyfluoroalkyl substances (PFAS) across the placental barrier between mother and fetus. Meconium samples were found to contain 25 PFAS, indicating their metabolism and excretion through meconium The excretion efficiency of perfluoroalkyl sulfonic acids shown greater removal by meconium compared to perfluorinated carboxylic acids [138].

Recent evidence indicated that short-chain PFAS are not toxicologically inert, despite their reduced biological half-lives and generally lower bioaccumulation potential compared with long-chain PFAS. Table 2 summarizes that repeated-dose animal studies have indicated quantifiable hepatotoxic effects for short-chain compounds such as PFBS and PFHxA, with defined NOAEL values based on liver-related endpoints. In parallel, emerging experimental studies in 2024–2025 (Office of Environmental Health Hazard Assessment [OEHHA] 139; Iulini et al. 140) demonstrate that short-chain PFAS can modulate immune cell function in vitro, suggesting immunotoxic potential under chronic or repeated exposure scenarios. Although their toxic potency is typically lower than that of legacy long-chain PFAS, the high environmental mobility and continuous exposure routes of short-chain PFAS warrant careful consideration in long-term risk assessment and regulatory decision-making.

## Regulatory landscape and risk assessment

### Current guidelines and thresholds

Multiple environmental media, such as water, soil, and food, have been assigned precise recommendations and threshold values by prominent regulatory bodies. The Environmental Protection Agency (EPA) in the United States established 70 parts per trillion (ppt) as the maximum allowed concentration of specific PFAS compounds in drinking water [141]. The Drinking Water Directive in Europe recommends drinking water standards at 100 nanograms per liter (ng/l) for single PFAS compounds and 500 ng/l for the combined total of 20 PFAS compounds (Wee & Aris, 2019). The United States has multiple states which have implemented surface water quality standards for PFOS and PFOA at different concentration levels from 0.0047 ng/l to 600 ng/l [142]. The lack of defined PFAS risk management techniques hinders effective risk control [143]. The Ministry of Environment together with environmental agencies has recognized the insufficient PFAS standards for water and soil and food monitoring. The United States Environmental Protection Agency has established Maximum Contaminant Levels (MCLs) for specific PFAS compounds including PFOA at 4 ng/l and PFOS at 4 ng/l and PFHxS and PFNA and Genx at 10 ng/l. The United States public water supply systems must follow these established regulations. The European Union drinking water standards establish 20 specific PFAS compounds at < 0.1 µg/l each and a combined PFAS limit at < 0.5 µg/l. The Canadian government has established a drinking water standard of 30 ng/L for 25 monitored PFAS compounds.

The Netherlands, Denmark, and Germany have instituted defined PFAS content limits in soil across their European regions. The Danish government has has set a PFOS limit in soil at 0.002 micrograms per kilogram (µg/kg) [144]. The United States has not established federal soil-specific PFAS regulations at the federal level so states have taken charge of creating their own regulations. The proposed threshold value of 0.01 mg/kg serves as a standard for biosolids applications in Denmark. The high PFAS concentrations in landfill leachate have forced local authorities to create treatment standards for this waste product. The permissible PFOS limits in freshwater and marine environments are 36 µg PFOS/l, whereas in biota samples, they are 7.2 µg PFOS/l and 9.1 µg PFOS/kg, respectively.

Numerous countries globally are advancing their own PFAS food laws; nevertheless, these standards remain inconsistent. Research indicates that processed foods may have PFAS pollutants due to packing materials and industrial processing; nevertheless, there are no standardized food safety regulations in place [145]. The European Food Safety Authority established a Tolerable Weekly Intake (TWI) of 4.4 ng/kg bw per week for total priority PFAS compounds (PFOA, PFOS, PFNA and PFHxS) to protect against immunological damage which serves as the foundation for European Union food risk evaluations. The United States establishes separate Maximum Contaminant Levels (MCLs) for multiple PFAS compounds (ng/l) but the EU uses a group-based system with 20 compounds and total PFAS at higher limits for each substance and Canada uses 25 compounds sum approach. The technical limits for PFAS compounds in soil and sludge differ between groups of substances. The European Food Safety Authority established a Tolerable Weekly Intake (TWI) for four PFAS compounds which serves as the foundation for food exposure assessments. Regulatory PFAS thresholds by region and compound are summarized in Table 4.

**Table 4:** Regulatory PFAS thresholds by region and compound

Region	Matrix	Compounds	Regulatory threshold	References
United States (EPA)	Drinking water	PFOA, PFOS	4 ng/L (ppt)	EPA [146]
United States (EPA)	Drinking water	PFHxS, PFNA, HFPO-DA (GenX), PFBS	10 ng/L	EPA [146]
European union – recast drinking water directive	Drinking water	PFAS	0.1 µg/L (100 ng/L)per summed 20 PFAS	EEA’s waterbase 147
European union – recast drinking water directive	Drinking water	PFAS	0.5 µg/L	EEA’s waterbase 147
Canada – Health Canada	Drinking water	PFAS	30 ng/L (sum of 25 PFAS)	(Canadian drinking water quality 148)
Denmark – Danish Guidance	Soil (sewage sludge)	PFAS	10 µg/kg	(Ministry of environment denmark 149)
Denmark – Danish guidance	Freshwaters & marine waters	PFOS	36 µg PFOS/L & 7.2 µg PFOS/L	(Ministry of environment denmark, 149)
Denmark – Danish guidance	Biota	PFOS	9.1 µg PFOS/kg	(Ministry of environment denmark, 149)
EU / EFSA (food risk assessment)	Dietery exposure (food)	PFOA, PFOS, PFNA, PFHxS	4.4 ng/kg	(Efsa 150)

### Challenges in setting safety limits

The scientific community encounters significant difficulty in determining safety thresholds for PFAS because to persistent ambiguities regarding their unique features and their varying harmful effects on diverse bodily systems at various developmental stages [151]. The diverse chemical composition of PFAS creates difficulties in understanding their health effects because each compound [152]. The insufficient scientific evidence about numerous PFAS compounds creates obstacles for both risk assessment and regulatory activities. The unknown scientific evidence about PFAS compounds creates difficulties for both researchers and regulators to determine their complete health and environmental effects.

The multitude of different PFAS chemicals is deficient in adequate toxicological data, posing a significant challenge for risk assessment [152, 153]. Scientists have conducted extensive research on PFOA and PFOS but most other PFAS compounds remain poorly understood [152]. The insufficient available data prevents scientists from determining the individual and combined risks of these compounds which hinders the creation of effective safety thresholds. The introduction of untested PFAS compounds into the environment perpetuates data deficiencies, prolonging regulatory delays [153].

The process of establishing safety limits becomes more complicated because PFAS exist as complex mixtures in environmental settings. Scientists struggle to link specific toxic effects to particular compounds because PFAS exist as combined substances in environmental samples [153, 154]. The current risk assessment systems for chemical mixtures lack effectiveness because they do not properly measure how different PFAS compounds interact with each other. The advancement of improved techniques for analyzing the consequences of PFAS mixtures is a pressing necessity for formulating superior regulatory decisions and public health guidelines [154].

### Gaps in global harmonization

Various nations and regions employ divergent regulatory approaches to set PFAS contamination thresholds. The European Union develops strict emission controls for PFAS because it recognizes the severe environmental and health risks from excessive accumulation [155]. The identification of PFAS poses challenges for African nations due to inadequate analytical equipment and substantial testing costs, resulting in weak and constrained regulatory frameworks [156]. The United States shows inconsistent PFAS regulation because different states have established their own specific biosolids contamination standards [144].

The world lacks a standardized definition for determining safe PFAS exposure thresholds. The U.S. Environmental Protection Agency (EPA) and European Union established drinking water guidelines through health advisory levels for PFOA and PFOS but these standards do not match between countries [157]. The different regulatory bodies maintain different standards for PFAS contamination in rainwater which results in conflicting safety thresholds [157]. The many characteristics of PFAS compounds complicate the establishment of a uniform regulatory standard, as each component possesses distinct properties and associated health hazards.

### Frameworks for ecological and human health risk assessment

The benchmark dosage approach allows researchers to determine the specific dose which produces a defined adverse effect response rate for systematic toxicity assessments. The study by [158] used benchmark concentrations (BMCs) to analyze PFAS potency in human liver spheroids through high-throughput transcriptomic analysis which enabled researchers to compare transcriptional changes with cytotoxicity effects between different compounds. The MoE approach enables researchers to evaluate PFAS compounds through the combination of bioactivity data with human biomonitoring results to determine their potential risks. The BCBCR calculation from [159] helps scientists determine which PFAS compounds need additional risk assessment through their blood concentration to bioactive concentration ratio. The screening method based on BCBCR values assists researchers in determining which PFAS chemicals require more assessment due to their reduced BCBCR values. The approach demonstrates efficacy in screening persistent chemicals, including PFAS.

The hazard index and relative potency factor methods represent new approaches to study PFAS mixtures through their analysis. The methods enable researchers to evaluate toxicological effects and potency levels between different PFAS compounds. The research shows that these methods enable risk assessment at screening levels by considering the specific dose-response patterns and chemical reaction mechanisms of PFAS compounds [160]. The scientific community has developed a tiered system for PFAS risk assessment because multiple PFAS compounds lack sufficient toxicity data. The multi-tiered assessment framework employs many forms of evidence, including combination toxicology and systematic dose-response analyses, due to the varied toxicological profiles of PFAS chemicals [153].

## Analytical methods for PFAS detection

### Sampling and pre-treatment challenges

The collection of PFAS faces major difficulties because of possible contamination that occurs when samples are gathered. PFAS exist in numerous consumer and industrial products which creates a broad risk for contamination. The use of fluoropolymers in standard sampling tools requires best practices to prevent cross-contamination according to Wanzek et al. [161]. The implementation of standardized procedures helps scientists achieve precise PFAS concentration measurements in environmental samples while reducing measurement errors. The identification of presumptive PFAS contamination sites through regular screening enables scientists to develop targeted sampling approaches [162]. The implementation of strict laboratory protocols regarding sample storage temperatures and hold times prevents PFAS from binding to containers and reduces their degradation [161]. The implementation of passive sampling devices represents a new technology which enhances PFAS detection accuracy while minimizing the risk of contamination. The use of graphene-based hydrogel monoliths and ceramic filter tubes provides reliable PFAS capture with low interference levels during field operations in the field compared to conventional grab sampling techniques [163].

PFAS analysis requires matrix-specific pretreatment due to distinct challenges in water, soil, and biological samples. Aqueous samples undergo filtration and solid-phase extraction (SPE) with WAX or HLB cartridges to concentrate PFAS and reduce interference. Soil and sediment samples need more intensive extraction using methanol or methanol–alkaline solvents with shaking, sonication, or accelerated solvent extraction (ASE), followed by cleanup with graphitized carbon black (GCB) or dispersive SPE to remove organic matter and lipids. Iannone et al. [164], Jia [165], Zhou [166] Matrix-specific differences in PFAS pretreatment strategies are summarized in Table 5.

**Table 5:** Matrix-specific pretreatment strategies for PFAS analysis

Sample matrix	Typical extraction solvent(s)	Pretreatment/cleanup steps	Key analytical challenges
Water (surface water, groundwater, drinking water)	Not applicable (direct extraction)	Filtration (0.45 μm), solid-phase extraction (SPE) using WAX or HLB cartridges	Low PFAS concentrations, background contamination, breakthrough of short-chain PFAS
Soil / Sediment	Methanol; methanol–alkaline mixtures	Shaking, sonication, or accelerated solvent extraction (ASE); cleanup using dispersive SPE or graphitized carbon black (GCB)	High organic matter content, co-extracted interferences, variable recovery
Biological samples (fish tissue, blood, breast milk)	Methanol or acetonitrile	Protein precipitation, enzymatic or alkaline digestion, followed by SPE purification	High lipid and protein content, ion suppression/enhancement, matrix effects

*SPE* solid-phase extraction, *WAX* weak anion exchange, *HLB* hydrophilic–lipophilic balance, *ASE* accelerated solvent extraction

Biological samples, including fish tissue, blood, and breast milk, necessitate pretreatment processes such as protein precipitation using acetonitrile or methanol, enzymatic digestion, or alkaline treatment. These are followed by solid-phase extraction (SPE) to mitigate ion suppression in LC-MS/MS analyses. In the absence of appropriate pretreatment, issues such as recovery bias, signal interference, and underestimation of short-chain PFAS may arise, underscoring the importance of employing matrix-specific extraction and purification techniques Mišľanová and Valachovičová [167].

### Detection techniques

The detection of PFAS occurs through targeted methods which include Liquid Chromatography-Tandem Mass Spectrometry (LC-MS/MS) and Gas Chromatography-Mass Spectrometry (GC-MS). The methods of LC-MS/MS and GC-MS serve as primary detection tools for PFAS because they provide high sensitivity and specificity to detect trace amounts of PFAS in environmental and biological samples. The technique of Liquid Chromatography-Tandem Mass Spectrometry (LC-MS/MS) stands as a leading method for PFAS detection because it delivers exceptional sensitivity and specificity. The method enables researchers to detect PFAS at extremely low concentrations which reach parts per trillion (ppt) levels. The method shows superior results for studying non-volatile PFAS compounds. The method uses liquid chromatography separation techniques with mass spectrometry analysis to detect PFAS compounds in complex sample mixtures [165]. The detection of PFAS through Gas Chromatography-Mass Spectrometry (GC-MS) remains less popular than LC-MS/MS because PFAS compounds show resistance to heat and lack sufficient volatility. The detection of volatile and semi-volatile PFAS compounds becomes possible through GC-MS when researchers perform derivatization to make these compounds volatile. The method shows excellent results for detecting specific PFAS compounds including fluorotelomer alcohols [168]. The detection limits of GC-MS remain higher than LC-MS/MS for non-volatile PFAS compounds but the method finds use in specific applications where its special features become useful. The detection limits of LC-MS/MS and GC-MS depend on the particular PFAS compound and the sample matrix being analyzed. The detection limits of LC-MS/MS reach below parts per trillion (ng/l) which fulfills the requirements of new regulatory standards [169]. The detection range of LC-MS extends from 0.01 to 0.08 ng/l in seawater and from 0.002 to 0.018 ng/g in marine sediments [164]. The method of LC-MS serves as the primary choice for PFAS analysis in water and soil samples because it delivers superior detection capabilities [170]. The detection of PFAS from environmental samples using GC-MS faces multiple analytical challenges. The thermal resistance of PFAS compounds makes GC-MS an infrequent choice for their detection. The detection range of GC-MS remains unclear because it needs extensive sample preparation procedures (Song, 2025).

Matrix effects play a significant role in PFAS detection and must be taken into account when comparing LC-MS/MS and GC-MS. In complex matrices like soils and biological samples, components such as organic matter, proteins, and lipids can lead to ion suppression or enhancement in LC-MS/MS, potentially skewing quantification. Consequently, rigorous cleanup procedures and the use of isotope-labeled internal standards are crucial to ensure analytical accuracy [171].

The effectiveness of analytical methods is heavily influenced by the physicochemical properties of PFAS and the sample matrix. LC-MS/MS is particularly well-suited for non-volatile and ionic PFAS, such as PFCAs and PFSAs, due to its high sensitivity and compatibility with aqueous extracts. Conversely, GC-MS is more appropriate for volatile and semi-volatile PFAS precursors, like fluorotelomer alcohols, typically following derivatization. Thus, employing matrix- and compound-specific analytical techniques is essential for minimizing matrix effects and ensuring reliable PFAS measurements [172–174].

### Emerging methods

The Total Oxidizable Precursor (TOP) assay is a crucial technique for transforming legacy PFAS precursors into identifiable end products, hence facilitating detailed investigations PFAS contamination. The TOP assay demonstrates success in detecting PFAS precursors within landfill leachate and groundwater samples which shows these precursors transform into identifiable PFAS compounds after prolonged environmental exposure [175].

PFAS analysis depends heavily on High-Resolution Mass Spectrometry (HRMS) because it provides exceptional resolution and precision which enables both targeted and non-targeted screening methods. The combination of ultra-high pressure liquid chromatography with Orbitrap mass spectrometry enables quantitative analysis and suspect and non-target screenings which show PFAS concentration reductions in particular populations have experienced [176]. The Fourier-transform ion cyclotron resonance mass spectrometry (FT-ICR MS) screening approach identifies novel PFAS chemicals in intricate mixtures by comprehensive analysis [177].

The Non-Targeted Screening method uses HRMS to scan for all PFAS compounds in samples without requiring known compound information which reveals new and developing PFAS substances. The combination of non-targeted analysis with suspect screening allows researchers to identify compounds through their distinctive fragmentation patterns and characteristic mass spectra. The method helps scientists discover unanticipated PFAS compounds in environmental samples which expands their knowledge about PFAS contamination [178].

### Limitations in detecting diverse PFAS compounds

**Fig. 5:**
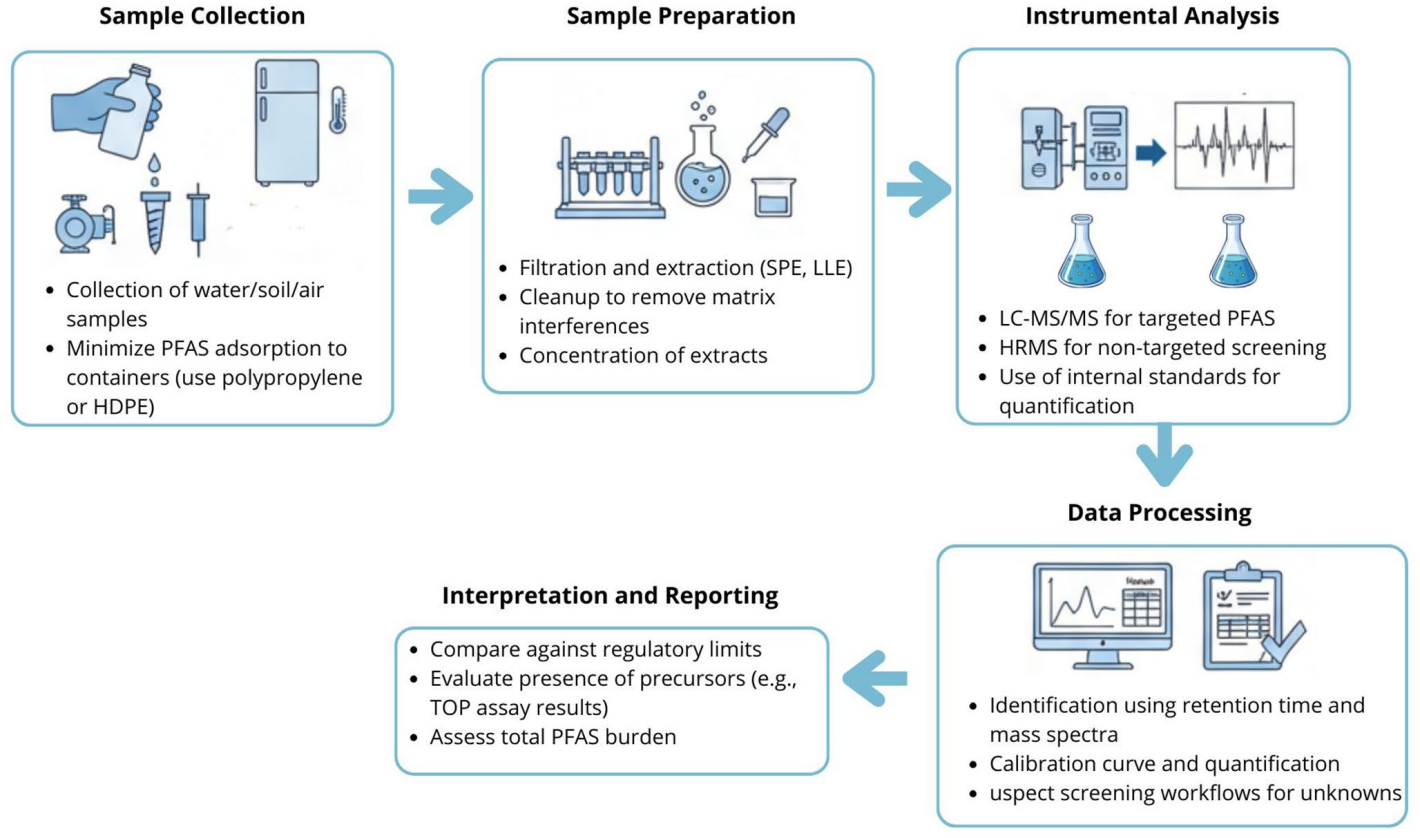
Workflow of PFAS analysis from sampling to quantification

The identification of various PFAS compounds becomes difficult because of matrix interference and unidentified precursors and insufficient analytical detection capabilities which result in poor quantification and identification results. The three environmental matrices of water and soil and air each introduce distinct analytical obstacles because of their background interference patterns. The extraction and detection performance of PFAS becomes inconsistent because organic matter and metals and other co-contaminants interfere with the analysis [164]. The extraction process of PFAS from samples through sample preparation methods can produce biased results because PFAS molecules bind to collection materials and preparation techniques create measurement errors that result in incorrect concentration readings [179]. The PFAS analysis workflow from sampling to quantification is shown in Fig. 5.

The detection of PFAS precursors faces significant obstacles because scientists cannot identify these compounds. The large class of diverse and evolving chemical structures includes thousands of compounds with different molecular structures. Scientists have not identified most of these compounds because they belong to novel derivatives and they appear in degradation pathways. The absence of complete databases and detection protocols for these compounds results in their inability to be detected [180]. The transformation of PFAS precursors into standard PFAS compounds during laboratory tests results in two possible outcomes: either the compounds remain unmeasurable or their concentration levels become misinterpreted. The total oxidizable precursor (TOP) assay detects precursor transformations but fails to detect all possible precursors [181].

The detection of PFAS compounds becomes less effective because of existing analytical gaps in their identification. The detection of PFAS at environmentally relevant levels requires analytical methods to operate at their maximum capacity. The current analytical techniques face difficulties in reaching the required sensitivity and specificity levels for PFAS detection through LC-MS/MS methods [169]. Nontargeted screening limitations develop comprehensive methods that can effectively screen for known and unknown PFAS in various matrices is ongoing. High-resolution mass spectrometry aids non-targeted analysis, but the vast number of potential compounds strains existing methodologies [182]. Table 6 compares analytical techniques for PFAS in terms of sensitivity, scope, and advantages.

**Table 6:** Comparison of analytical techniques for PFAS: sensitivity, scope, advantages

Technique	Scope	Typical LOD	Sensitivity	Typical application	Advantages	Representative references
Targeted Analysis, LC-MS/MS	Specific, typically a small set (tens) of known PFAS	0.1–1 ng/L (ppt) in water; 0.1–1 ng/g in solids	High (low parts-per-trillion or ppt)	Routine regulatory monitoring, compliance testing, drinking water analysis (e.g., EPA Methods 537.1, 533)	High sensitivity and specificity for target compounds, accurate quantification using authentic standards, established regulatory methods.	US EPA [146, 183]; Schymanski et al. [31]
GC-MS/MS	Volatile & semi-volatile PFAS (e.g., FTOHs)	1–10 ng/L (after derivatization)	Moderate to High (improving)	Air samples, precursor analysis, source identification	Suitable for neutral/volatile PFAS; good chromatographic separation	Butt et al. [184]
Non-Targeted/Suspect Screening (e.g., LC-HRMS/Q-TOF)	Broad, potentially thousands of known and unknown PFAS	1–10 ng/L (compound-dependent)	Moderate to High (improving)	Unknown compound identification, novel PFAS discovery, comprehensive environmental profiling.	Identifies unknown and emerging PFAS not covered by targeted methods, allows for retrospective data analysis.	Schymanski et al. [31]; Kwiatkowski et al. [185]
Total Oxidizable Precursor (TOP) Assay	Measures total oxidizable PFAS precursors	~ 5–50 ng/L (as PFCA equivalents)	Variable	Environmental samples to determine the potential concentration of precursors that can transform into terminal PFAS.	Provides an estimate of the total potential burden of PFAS in a sample, including precursors not measured by targeted methods.	Houtz and Sedlak [186]; Mejia-Avendaño et al. [187]
Total Organic Fluorine (TOF) or Extractable Organic Fluorine (EOF)	Measures total (or extractable) fluorine from all organofluorine compounds	~ 0.5–5 µg/L	Low to Moderate (microgram/L or parts-per-billion)	Screening to determine the presence of significant unidentified organofluorine (the “PFAS gap”).	Simple screening tool to assess the overall level of fluorinated contaminants, helpful in the Fluorine Mass Balance (FMB) approach.	Miyake et al. (2019); Sadia et al. [62]

LOD values represent typical ranges reported in the literature and regulatory methods; actual sensitivity depends on matrix, cleanup efficiency, and instrument configuration

Despite significant advancements in PFAS monitoring and risk assessment, several limitations still hinder accurate exposure characterization and risk evaluations. Current monitoring programs mainly depend on targeted analytical methods that capture only a limited subset of known PFAS, potentially missing numerous precursors and transformation products. Additionally, matrix effects and varying pretreatment efficiencies across water, soil, and biological samples introduce uncertainties in quantification, especially for short-chain PFAS present at low concentrations. Risk assessment frameworks face further challenges due to the scarcity of compound-specific toxicological data for many emerging and short-chain PFAS, along with a limited understanding of mixture effects under chronic low-dose exposure scenarios. As a result, regulatory thresholds often rely on a small number of well-studied legacy compounds, which may not fully represent cumulative or class-based risks. These constraints highlight the necessity for expanded analytical scope, improved integration of non-target screening, and more extensive toxicological and epidemiological data to facilitate rigorous and standardized PFAS risk evaluations.

## Remediation and treatment technologies

### Water treatment approaches

The physical removal procedures comprise three methods: activated carbon adsorption and membrane filtration and coagulation. Activated carbon in its powdered form (PAC) demonstrates strong ability to remove PFAS compounds including perfluorooctanoic acid (PFOA) and perfluorooctanesulfonic acid (PFOS). The addition of polymer-stabilized PAC to water treatment systems creates an effective barrier that enhances PAC sorptive capacity [188]. The removal of PFAS becomes possible through Membrane Filtration techniques which include reverse osmosis and nanofiltration (NF). The combination of NF with foam fractionation of concentrate and co-surfactants leads to high PFAS removal efficiency. The treatment method achieves more than 98% PFAS concentration decrease in treated water [189]. The addition of metal coagulants including aluminum and iron to water treatment processes enables PFAS removal through floc-based adsorption. The efficacy of removal is contingent upon the selection of coagulant and the operational parameters, including pH levels and dosages of the coagulant [190].

The adsorption of PFAS onto activated carbon encompasses both physical and chemical mechanisms rather than a single nonspecific process. Physical adsorption is mainly driven by hydrophobic interactions and van der Waals forces between the fluorinated carbon chain of PFAS and carbonaceous surface. This mechanism is particularly effective for long-chain PFAS, which exhibit stronger hydrophobicity and higher affinity for microporous carbon structures [191].

Chemical adsorption, although typically less prevalent, can occur through the interaction of PFAS functional head groups, such as carboxylate or sulfonate moieties, with surface functional groups on activated carbon, including hydroxyl, carbonyl, and amine groups. The presence and arrangement of these surface functionalities affect electrostatic interactions and hydrogen bonding, which in turn influence adsorption selectivity, particularly for short-chain PFAS that are less hydrophobic and more challenging to eliminate [192].

The effectiveness of activated carbon in adsorption is greatly affected by its physicochemical characteristics, including pore size distribution, surface area, and surface chemistry. Activated carbon, with its high surface area and microporous structure, is particularly effective at eliminating long-chain PFAS. In contrast, mesoporous structures and tailored surface functionalization improve the accessibility and retention of short-chain PFAS. These structure–activity relationships explain the reduced adsorption efficiency of short-chain PFAS and highlight the importance of optimizing materials when employing activated carbon for PFAS remediation [193].

The Chemical Removal Techniques consist of two methods which include electrochemical treatment and Advanced Oxidation Processes (AOPs). The electrochemical treatment methods of electrocoagulation and electrooxidation demonstrate ability to transform PFAS compounds into safer breakdown products. These are particularly suitable for treating water treatment residuals that contain concentrated PFAS [194]. Advanced Oxidation Processes (AOPs) involve the generation of highly reactive species like hydroxyl radicals that can break down PFAS. AOPs are often used in conjunction with other treatment methods to enhance overall PFAS degradation [195]. The performance of PFAS water treatment technologies under various conditions is summarized in Table 7.

**Table 7:** Performance of PFAS water treatment technologies under various conditions

Technology	Mechanism/process	Performance Under Various Conditions	Limitations/challenges	References
Activated carbon adsorption (GAC)	Adsorption of PFAS molecules onto activated carbon surface	Effective for a broad range of PFAS; performance influenced by seasonal variations affecting water production and influent concentration	Reduced efficiency at high PFAS loads; requires periodic regeneration or replacement	Jonathan et al. [196]
Membrane filtration (RO/NF)	Physical separation using semi-permeable membranes	Excellent PFAS removal efficiency across multiple chain lengths	Produces concentrated waste streams that require further treatment; high operational costs	Tow et al. [197]
Electrochemical treatments (electrocoagulation/electrooxidation)	Electrochemical degradation or coagulation of PFAS molecules	Promising under laboratory conditions with high PFAS concentrations	Less efficient at low, environmentally relevant concentrations; energy-intensive	Ryan et al. [194]
Electron beam technology	High-energy electrons break C–F bonds for PFAS degradation	Effective for PFAS destruction; suitable for concentrated streams	High energy demand; limited scalability for large flow systems	Londhe et al. [198]
Fluorine–fluorine interaction sorbents	Selective adsorption via F–F interactions between PFAS and fluorinated materials	High selectivity and efficiency even in presence of competing organic substances	Novel technology; requires optimization for field-scale use	Fu et al. [199]
Advanced oxidation and reduction processes (AOPs/ARPs)	Oxidative or reductive degradation via radicals or photochemical reactions	Variable performance depending on oxidant type, pH, and light intensity	Often high energy requirements; incomplete mineralization in some cases	Nzeribe et al. [200]
Integrated or hybrid systems	Combination of multiple treatment processes	Enhances robustness and adaptability across PFAS types and concentrations	Requires optimization of operational parameters; higher system complexity	Benaafi and Bafaqeer [195]

Numerous water treatment technologies have been suggested for removing PFAS, but their levels of technological advancement and readiness for practical application differ greatly. Among these, adsorption techniques, especially those utilizing granular activated carbon (GAC) and ion-exchange resins, are the most developed and commonly used on a large scale, particularly for long-chain PFAS. However, their efficiency diminishes when dealing with short-chain PFAS, and the need for frequent media replacement poses a significant operational challenge. On the other hand, advanced oxidation and reduction methods, such as electrochemical oxidation and plasma-based treatments, have demonstrated promising degradation results in laboratory and pilot-scale experiments. Nevertheless, they are constrained by high energy requirements and uncertain transformation pathways in real-world conditions [191, 201].

Recent research trends are increasingly concentrating on destructive treatment technologies, such as electrochemical mineralization and catalytic systems (e.g., MOF-based and carbon-based catalysts), which are designed to cleave the C–F bond rather than transferring PFAS to secondary waste streams. While these methods present substantial long-term benefits, their scalability, cost-effectiveness, and byproduct formation necessitate further validation through extended field trials.

Although numerous PFAS remediation technologies have been documented, their effectiveness varies significantly when assessed using quantitative performance metrics. Adsorption-based methods, such as granular activated carbon (GAC) and ion-exchange resins, typically achieve 70–99% removal of long-chain PFAS in full-scale water treatment systems. However, breakthrough of short-chain PFAS often occurs, and effluent concentrations may remain above emerging regulatory limits without frequent media replacement.

Technologies that utilize membranes, such as nanofiltration and reverse osmosis, are capable of rejecting more than 95% of PFAS across various chain lengths, often resulting in treated water that complies with strict guideline standards. However, these methods produce concentrated waste streams and require significant capital and energy investments, which restrict their use for large-scale or decentralized treatment applications.

Destructive technologies, including electrochemical oxidation, plasma treatment, and advanced reduction processes, have shown PFAS degradation rates of over 80–99% in both laboratory and pilot-scale settings, with some systems achieving nearly complete defluorination. Nevertheless, the reported treatment efficiencies are highly contingent on factors such as water chemistry, energy input, and reactor design. Additionally, long-term field data that consistently demonstrate compliance with regulatory thresholds remain scarce.

In the short term, adsorption and membrane technologies are currently the most dependable methods for meeting regulatory standards. Meanwhile, destructive techniques are emerging as a significant area of research, with the potential to offer sustainable long-term solutions once issues related to scalability, cost, and by-product generation are effectively resolved.

### Soil and groundwater remediation

**Fig. 6:**
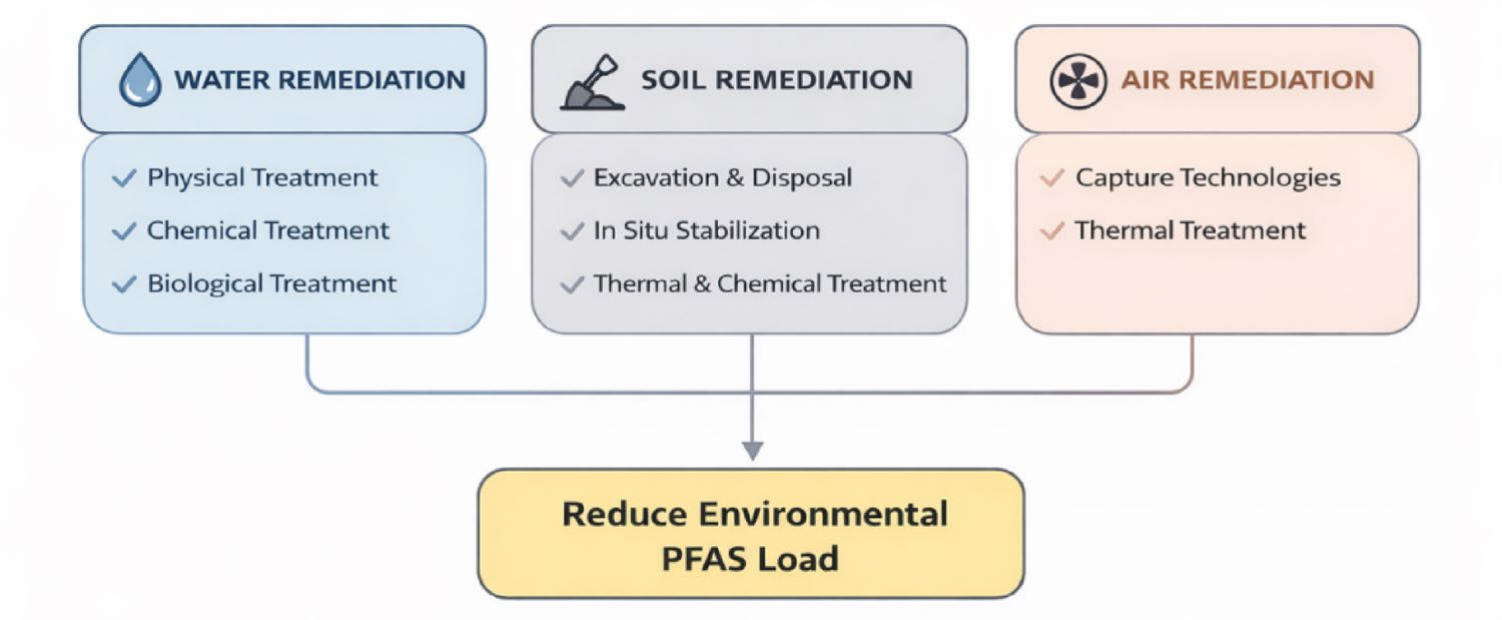
Flowchart of PFAS remediation pathways across media (water, soil, air)

Remediation of PFAS-contaminated soil and groundwater is critical due to their persistence and environmental impact. Various conventional and emerging methods are available to treat the contaminant (Fig. 6). The conventional methods decided into three different types. Excavation and disposal involve physically removing contaminated soil and disposing of it in landfills. While effective in isolating contamination, it does not treat PFAS and has high costs and environmental impacts. Activated Carbon Adsorption is a widely used adsorbent for PFAS removal from water. It captures PFAS through adsorption, but regeneration and disposal of the spent carbon present challenges. Pump and Treat Systems to pumped the groundwater to the surface and treated to remove contaminants up to 99% depend on treatment duration and media used, often using activated carbon or ion exchange resins. However, these systems can be energy-intensive and costly over long durations.

Emerging methods are important to treat the soil contaminant quickly. The process of bioremediation depends on microbial communities to break down contaminants. Scientists now study omics-based methods to discover better ways for soil microbes to eliminate PFAS contaminants in their natural environment [202]. The combination of Nano-technology with Synthetic Biology enables scientists to create new remediation methods which use engineered microorganisms and nanomaterials to precisely target and break down PFAS [203]. Scientists develop new adsorbents through research of metal-organic frameworks (MOFs) and graphene-based composites and biochar which demonstrate superior PFAS binding properties [204]. Scientists study Advanced Oxidation Processes (AOPs) including photocatalysis and sonolysis to understand their PFAS degradation potential through radical production but they face challenges with power consumption and large-scale implementation. Thermal Treatment uses high temperatures to transform PFAS compounds found in contaminated soil. Scientists investigate new methods of microwave and cryogenic treatment to enhance both thermal treatment efficiency and energy consumption [205].

In the field of soil remediation, physical removal and containment strategies, such as excavation and off-site disposal, continue to be the most dependable methods for addressing heavily contaminated sites, notwithstanding their substantial economic and environmental costs. Stabilization and immobilization techniques, including the use of carbonaceous amendments and biochar, have garnered attention as more cost-effective alternatives. Nevertheless, their efficacy is predominantly confined to reducing the mobility of PFAS rather than achieving complete destruction. Thermal treatment and soil washing can attain high removal efficiencies; however, they are limited by energy demands and the feasibility specific to each site [206, 207].

Current research hotspots focus on in situ remediation methods that blend adsorption and degradation mechanisms, alongside hybrid strategies that merge soil washing with destructive downstream treatments. These methods aim to balance remediation efficiency, cost, and environmental sustainability. However, most remain at the laboratory or pilot scale, underscoring the need for systematic field-scale performance evaluation [208, 209].

### Destruction and mineralization methods

The development of sophisticated methods to break PFAS bonds involves both destruction and mineralization approaches which address the strong carbon-fluorine bonds found in these substances. Advanced Reduction Processes (ARPs) employ reactive species including hydrated electrons (eaq-) to perform PFAS degradation. The UV/sulfite and UV/iodide systems represent notable degradation systems which breaks down different PFAS compounds through specific reaction pathways that depend on their head group and chain length structures [210]. For instance, the UV222/sulfite ARP system achieves approximately 85% reduction in perfluorooctyl sulfonic acid (PFOS) and 66% defluorination within 6 h, though shorter-chain PFAS show slower degradation [211]. The process of defluorination becomes more effective when UV/persulfate oxidation serves as a pretreatment method to remove contaminants that block the process by over 90% within 24 h [212]. The Low-Temperature Base-Mediated Mineralization process uses a solvent mixture with NaOH at mild temperatures to break carbon-fluorine bonds in fluorotelomers. The method proves effective for treating PFAS compounds with C-H bonds and works on both current PFAS products and degradation products from other treatment methods (Monsky et al. 2024). Thermal Treatment Techniques enhance PFAS mineralization through the addition of granular activated carbon (GAC) or noble metals which boost degradation rates [213]. The process of lower temperature degradation produces products of incomplete destruction (PIDs) when the degradation process is not fully completed (Shields et al. 214). The photocatalytic degradation process uses high-energy gap semiconductor oxides to perform PFAS mineralization through solar-powered reactions. The experimental stage of this technology seeks to harness renewable energy sources for PFAS destruction [215]. Plasma-Based Technology produces reactive species which demonstrate better degradation efficiency than thermal methods while using less energy. The development of these technologies needs additional work to achieve operational readiness [200].

## Innovations and emerging solutions

### PFAS-free product development

The lithium-ion battery industry shows a major development in energy storage technology. Scientists work to create PFAS-free materials which will replace PFAS in electrode and electrolyte applications. Multiple companies now work on developing PFAS-free materials for electrodes and electrolytes. The production of PFAS-free batteries remains possible although these solutions have not reached commercial status. The battery industry must achieve environmental sustainability through performance optimization of new components which represents a fundamental transformation in battery development [216].

The firefighting industry focuses on replacing aqueous film-forming foams (AFFF) containing PFAS with fluorine-free alternatives. The military adopted a new specification for fluorine-free foam during 2023 because it represents a transition toward environmentally friendly solutions. The transition process requires detailed evaluation of performance levels and financial costs and regulatory standards to create safer and more sustainable fire suppression systems [217, 218]. The online database contains detailed information about PFAS applications and shows 530 fluorine-free alternatives for different industries but some industries need additional substitute materials. The database helps businesses locate suitable PFAS alternatives for their operations which enables them to adopt environmentally friendly manufacturing methods [219].

### Green chemistry alternatives

The development of green chemistry alternatives by industries and researchers focuses on creating degradable compounds which match the functional properties of PFAS. The green chemistry alternatives for PFAS degradation include enzymatic breakdown and biodegradable polymer use and microbial cleanup methods. Research into enzymatic degradation shows promise because scientists study how extracellular enzymes can break down PFAS compounds. The enzymes operate as environmentally friendly solutions because they break down naturally and they need minimal power to function ([220]; X. Li et al. 221). Research on enzyme-based PFAS degradation shows progress but scientists predict future studies will improve enzyme-based PFAS breakdown methods [222]. The removal of PFAS occurs through adsorption and catalytic degradation when scientists use zeolites and metal-organic frameworks (MOFs) and covalent organic frameworks (COFs) as functional framework materials. The sustainable PFAS removal technology represents a major advancement in environmentally friendly remediation systems (X. Li et al. 221). Scientists investigate biodegradable polymers including polylactic acid (PLA) and polybutylene succinate for use as PFAS alternatives in food packaging applications. The materials combine PFAS-like barrier properties with environmental protection goals [223]. Scientists continue to study microbial remediation through the development of specific enzymes and microbes which can transform PFAS compounds. Scientists study haloacid dehalogenases and cytochromes P450 enzymes because they show promise for PFAS breakdown [92]. Green Chemistry Principles focus on creating environmentally friendly chemical manufacturing methods through sustainable production processes. The design of environmentally friendly compounds requires scientists to use sustainable materials and develop efficient production methods which generate minimal waste [224].

### Biodegradation and enzymatic pathways

Biological and bioprocess-based remediation strategies for per- and polyfluoroalkyl substances (PFAS) are gaining attention as sustainable alternatives to energy-intensive physicochemical treatments. Microbial degradation relies on specific bacteria and fungi capable of mediating reductive, oxidative, or hydrolytic transformations of PFAS, including strains of *Pseudomonas* and *Acidimicrobium* reported to induce partial defluorination under controlled conditions [225, 226]. However, confirmed PFAS-degrading microbial diversity remains limited, and complete mineralization is rarely achieved. Ongoing research focuses on metagenomic screening, adaptive enrichment, and synthetic biology approaches to enhance catalytic efficiency and substrate specificity [88, 92].

Enzymatic pathways further support PFAS transformation through enzymes such as haloacid dehalogenases, cytochromes P450, reductive dehalogenases, laccases, and peroxidases, which facilitate C–F bond cleavage via electron transfer and radical-mediated mechanisms (Harris et al. 2024; [227]). Emerging strategies including extracellular enzyme systems, in silico protein engineering, and integration with bioelectrochemical systems have demonstrated promising laboratory-scale results. Nevertheless, kinetic limitations, fluoride release toxicity, environmental variability, and scale-up challenges remain significant barriers to field deployment, highlighting the need for continued interdisciplinary innovation to achieve practical and sustainable PFAS biodegradation.

### Nanomaterials and novel sorbents

The development of advanced materials which demonstrate strong sorption abilities and catalytic properties for PFAS removal from environmental sources represents a critical solution to their extended persistence and toxic effects. Fluorinated Sorbents (Fluorosorbents) achieve better PFAS removal through their strong fluorine-fluorine (F···F) bonding interactions which improve their selectivity and removal efficiency. The removal efficiency of Fluorosorbents exceeds other sorbents when operating under competitive conditions. The F–F bonding mechanism in Fluorosorbents enables them to establish stronger chemical bonds with PFAS compounds [199]. The porous structure of Metal-Organic Frameworks (MOFs) enables targeted post-synthetic modifications which makes them suitable for PFAS adsorption. The Zr-MOF TFA-MOF-808 shows exceptional PFAS adsorption performance because it uses both coordinative bonding and hydrophobic interactions. The material shows exceptional performance for both short-chain and long-chain perfluorinated carboxylic acid adsorption [228]. The design of Magnetic Fluorinated Polymer Sorbents enables efficient PFAS capture and allows for magnetic recovery during the regeneration process. The sorbents demonstrate better PFAS removal performance than conventional activated carbon and ion-exchange resins while showing fast adsorption rates [229]. The sorption of perfluorooctanoic acid (PFOA) and other PFAS compounds from water occurs effectively through Amphiphilic Perfluoropolyether Copolymers. The sorbents demonstrate high specificity for PFAS binding even when biomolecules are present in complex matrices [230]. The PFAS sorption capabilities of Carbon-Rich Materials depend on their CORG/O molar ratio and specific surface area measurements. The materials use hydrophobic bonding to achieve strong PFAS adsorption [231]. The development of nanomaterials and sorbents for PFAS remediation has created essential new methods to combat environmental and health risks from these persistent compounds.

### Policy-driven innovations and circular economy approaches

**Fig. 7:**
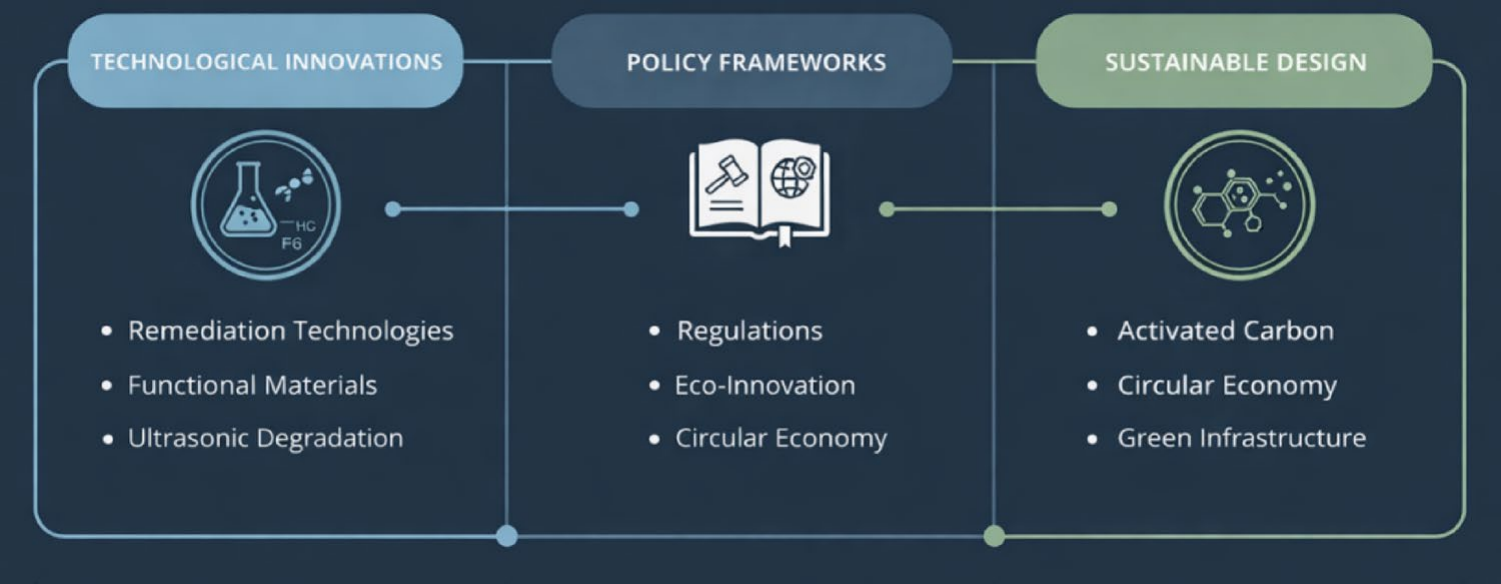
Innovation landscape linking technology, policy, and sustainable design

The implementation of PFAS reduction through sustainable production and consumption models becomes more effective when circular economy principles are integrated (Fig. 7). The resource-efficient waste-minimizing closed-loop systems of circular economy models can be designed to handle PFAS contamination effectively. The 6R strategy (Reduce, Reuse, Repair, Refurbish, Repurpose, and Recycle) of circular economy frameworks and sustainable production systems enables sustainable consumption and production models [232]. The Sustainable Development Goal 12 promotes responsible consumption and production through circular methods which help industries reduce their environmental footprint. The achievement of zero waste sustainability requires businesses to make essential changes to their supply chain operations [233]. The implementation of circular design principles and resource optimization enables businesses to decrease waste generation while developing environmentally friendly manufacturing methods which include PFAS emission reduction and contamination prevention. The EU’s Circular Economy Action Plan serves as a policy framework which enables businesses to adopt circular models through green taxation incentives [234]. The implementation of such policies enables businesses to integrate PFAS reduction methods into their existing sustainable operations. Remediation and technological innovation PFAS contamination in waste management requires advanced remediation technologies to stop PFAS from reaching the environment [87]. A circular economy model that enhances sustainability depends heavily on advanced waste management technologies including composting systems. The construction industry along with other sectors need customized approaches to adopt circular economy principles. The construction industry achieves substantial cost savings through its commitment to material recycling and reuse practices [235]. An overview of emerging PFAS alternatives and innovative mitigation strategies is provided in Table 8. The implementation of these principles helps decrease PFAS contamination in products and waste materials which results in better sustainability outcomes. Regulatory thresholds cited in this review reflect the most recent guidance available at the time of manuscript revision; readers are encouraged to consult official regulatory sources for future updates, as PFAS regulations continue to evolve.

**Table 8:** Summary of emerging PFAS alternatives and mitigation innovations

Category	Description	References
PFAS alternatives	Due to the environmental and health concerns associated with legacy PFAS, researchers are identifying alternative substances. New PFAS compounds have been introduced, though their environmental impacts still require further evaluation.	Kirkwood-donelson et al. [172]
Mechanochemical treatment	A process that uses mechanical force to induce chemical reactions, effectively degrading PFAS compounds.	Berg et al. [236]
Electrochemical oxidation	Utilizes electrical currents to break down PFAS, converting them into less harmful compounds.	Berg et al. [236]
Gasification and pyrolysis	Thermal processes that decompose PFAS at high temperatures in oxygen-poor environments, breaking the strong carbon-fluorine bonds.	Berg et al. [236]
Supercritical water Oxidation	Treats PFAS-contaminated wastes under high pressure and temperature in supercritical water, resulting in effective decomposition.	Berg et al. [236]
Regulatory and research efforts	Global regulatory agencies, including the U.S. EPA, are developing new standards and promoting research to improve PFAS mitigation strategies.	Hatton and Holton [237], Meegoda [238]
Analytical methods	High-resolution mass spectrometry methods have been developed to detect and characterize unknown PFAS compounds, providing insights into their environmental behavior and supporting new mitigation strategies.	Kirkwood-donelson et al. [172]

## Conclusions

PFAS constitute a considerable environmental and public health issue owing to its persistence, mobility, and intricate toxicological characteristics. While existing studies have improved understanding of PFAS sources, fate, detection, and remediation, important knowledge gaps remain, particularly for short-chain PFAS. Future research should prioritize the development of more sensitive and standardized analytical methods for detecting short-chain PFAS in complex matrices, as well as the field-scale evaluation of emerging remediation technologies such as MOF-based adsorbents to assess their long-term performance and sustainability beyond laboratory conditions. In parallel, greater regulatory harmonization and group-based PFAS management approaches are needed to align scientific advances with policy implementation and to enable more effective, coordinated mitigation of PFAS contamination at regional and global scales. Despite significant advances in understanding the occurrence, toxicity, and remediation of PFAS, several priority research directions remain crucial for future progress. First, analytical methods need further optimization for short-chain and emerging PFAS, particularly to enhance detection sensitivity, minimize matrix effects, and enable comprehensive non-target screening across diverse environmental matrices. Second, although many emerging remediation technologies, such as electrochemical oxidation, plasma treatment, and MOF-based adsorption and catalytic systems, have demonstrated promising laboratory-scale performance, their long-term effectiveness, stability, and cost efficiency under field conditions remain largely unvalidated. This underscores the need for extended pilot- and full-scale trials. Future research should prioritize toxicological studies that focus on specific compounds and mixtures, with particular attention to chronic low-dose exposure, immunotoxicity, and vulnerable populations. Moreover, a more profound integration of scientific research and regulatory development is essential to align risk assessment frameworks and facilitate evidence-based policy decisions. Addressing these research areas is crucial for enhancing sustainable PFAS management and alleviating long-term threats to human health and the environment.

## Data Availability

The data that support the findings of this study are available on request from the corresponding author. The data are not publicly available due to privacy or ethical restrictions.
